# Targeting TMPRSS2 and Cathepsin B/L together may be synergistic against SARS-CoV-2 infection

**DOI:** 10.1371/journal.pcbi.1008461

**Published:** 2020-12-08

**Authors:** Pranesh Padmanabhan, Rajat Desikan, Narendra M. Dixit

**Affiliations:** 1 Clem Jones Centre for Ageing Dementia Research, Queesnsland Brain Institute, The University of Queensland, Brisbane, Australia; 2 Department of Chemical Engineering, Indian Institute of Science, Bengaluru, India; 3 Centre for Biosystems Science and Engineering, Indian Institute of Science, Bengaluru, India; UNSW Australia, AUSTRALIA

## Abstract

The entry of SARS-CoV-2 into target cells requires the activation of its surface spike protein, S, by host proteases. The host serine protease TMPRSS2 and cysteine proteases Cathepsin B/L can activate S, making two independent entry pathways accessible to SARS-CoV-2. Blocking the proteases prevents SARS-CoV-2 entry *in vitro*. This blockade may be achieved *in vivo* through ‘repurposing’ drugs, a potential treatment option for COVID-19 that is now in clinical trials. Here, we found, surprisingly, that drugs targeting the two pathways, although independent, could display strong synergy in blocking virus entry. We predicted this synergy first using a mathematical model of SARS-CoV-2 entry and dynamics *in vitro*. The model considered the two pathways explicitly, let the entry efficiency through a pathway depend on the corresponding protease expression level, which varied across cells, and let inhibitors compromise the efficiency in a dose-dependent manner. The synergy predicted was novel and arose from effects of the drugs at both the single cell and the cell population levels. Validating our predictions, available *in vitro* data on SARS-CoV-2 and SARS-CoV entry displayed this synergy. Further, analysing the data using our model, we estimated the relative usage of the two pathways and found it to vary widely across cell lines, suggesting that targeting both pathways *in vivo* may be important and synergistic given the broad tissue tropism of SARS-CoV-2. Our findings provide insights into SARS-CoV-2 entry into target cells and may help improve the deployability of drug combinations targeting host proteases required for the entry.

## Author summary

The COVID-19 pandemic has triggered urgent efforts to repurpose available drugs for the treatment of SARS-CoV-2 infection. Synergistic drug combinations are particularly desirable because they allow the achievement of desired efficacies with minimal toxicities. Here, we predict that drugs targeting the host proteases, TMPRSS2 and Cathepsin B/L, which facilitate SARS-CoV-2 entry into cells through independent pathways, would be synergistic. We identified the synergy using a mathematical model and found evidence for it in available *in vitro* data. We found the estimated usage of the two pathways to vary vastly across cell types, highlighting the need to target both pathways. Our findings would help maximize the impact of combination therapies targeting host proteases involved in SARS-CoV-2 entry, which are currently in clinical trials.

## Introduction

As of August 13, 2020, the severe acute respiratory syndrome coronavirus 2 (SARS-CoV-2) had infected a total of over 20 million people and caused over 744,000 deaths across the world, starting from the first reported infections in Wuhan, China in December, 2019 [1]. SARS-CoV-2 is the third of the major coronavirus outbreaks this century, following SARS coronavirus (SARS-CoV) in 2002–03 and Middle East respiratory syndrome coronavirus (MERS-CoV) in 2012–13 [2]. The scale of the current pandemic far exceeds the earlier two and calls for urgent interventions. At the time of writing, no approved drugs or vaccines existed for any of these coronaviruses [3,4]. Massive efforts are ongoing to ‘repurpose’ drugs approved for unrelated conditions that may have an effect on SARS-CoV-2 [5–9]. For instance, remdesivir, a drug active against SARS-CoV and MERS-CoV, has been approved for limited use in severe cases of SARS-CoV-2 infection [10]. The steroid dexamethasone has also been approved for use in severe infections [11]. The anti-influenza drug favipiravir has been approved, on the other hand, for use in moderate infections [12]. The anti-malarial drug hydroxychloroquine, which has shown some activity against SARS-CoV-2 *in vitro*, has been approved in some countries as a prophylactic for high risk groups such as healthcare providers [13,14], although recent studies have questioned its ability to prevent infection of lung cells [15,16]. Several such drugs are under clinical trials, including type I interferons and the HIV-1 drugs lopinavir and ritonavir [17]. Simultaneously, efforts are underway to develop effective vaccines [4,18].

An important class of drugs under investigation putatively targets host factors involved in the lifecycle of SARS-CoV-2, which are being identified through exhaustive virus-host interactome maps [7,19]. Within this class, drugs that block SARS-CoV-2 entry into target cells may be particularly promising, for they would protect cells from becoming infected by the virus and may thus minimize disease. SARS-CoV-2 entry requires the binding of its spike protein, S, to the host cell surface receptor angiotensin-converting enzyme 2 (ACE2) [20,21]. In addition, S must be cleaved by host proteases, as with other coronaviruses [22] and influenza viruses [23], for successful entry, a step often limiting the zoonotic potential of coronaviruses [24]. *In vitro* studies have suggested that this cleavage, also termed S protein activation, can be accomplished by the transmembrane serine protease TMPRSS2 and endosomal cysteine proteases Cathepsin B and Cathepsin L [21,25]. These proteins have also been implicated in the entry of SARS-CoV [26–30], to which SARS-CoV-2 is closely related [31], and have formed the basis for corresponding studies on SARS-CoV-2. (MERS-CoV uses a different receptor, dipeptidyl peptidase 4, but appears to use the same proteases for activation [32,33].) ACE2, TMPRSS2 and Cathepsin B/L, thus, are key host targets for blocking SARS-CoV-2 entry. ACE2 plays a critical role in regulating several vital parameters, such as blood pressure, and targeting it could come with serious risks [34]. Targeting host proteases appears to have fewer side effects. Indeed, camostat mesylate, a drug that targets TMPRSS2, has been approved for use in the treatment of chronic pancreatitis and postoperative reflux esophagitis in Japan [35]. A human clinical trial has now been initiated to assess the possibility of using camostat mesylate monotherapy for treating SARS-CoV-2 infection (ClinicalTrials.gov ID: NCT04321096). Nafamostat mesylate, another drug targeting TMPRSS2 and approved in Japan as well as by the FDA, blocked SARS-CoV-2 entry more potently than camostat mesylate *in vitro* [36] and is also under clinical trials (ClinicalTrials.gov ID: NCT04352400). Drugs targeting Cathepsin B/L are under development [37,38]. Hydroxychloroquine is thought to act against SARS-CoV-2 via several mechanisms, including blocking the endocytic pathway; it suppresses clathrin-mediated virus uptake and also prevents endosomal acidification, the latter required for Cathepsin B/L activity [3,16,39]. While studies suggest that hydroxychloroquine monotherapy may be inadequately efficacious [15,16], its efficacy in combination with camostat mesylate is under clinical evaluation (ClinicalTrials.gov ID: NCT04338906).

The proteases TMPRSS2 and Cathepsin B/L are unrelated and work independently [22], suggesting that SARS-CoV-2 can enter cells via two independent pathways. Studies on SARS-CoV have shown that TMPRSS2 is expressed on the target cell surface and acts by cleaving S and facilitating the fusion of viral and cell membranes at the target cell surface, whereas Cathepsin B/L are expressed in endosomes and act after the virus has been endocytosed, facilitating the fusion of viral and endosomal membranes [26–30]. It appears, thus, that blocking either TMPRSS2 or Cathepsin B/L may be insufficient to block virus entry; the virus could continue to enter cells via the pathway that remains unblocked. Indeed, *in vitro* studies have shown, both with SARS-CoV and SARS-CoV-2, that simultaneous targeting of both TMPRSS2 and Cathepsin B/L is required for fully stopping virus entry [21,28]. The pathways have not been studied in SARS-CoV-2 infection *in vivo*.

Given the clinical trials underway with drugs blocking SARS-CoV-2 entry, we were interested in assessing whether combination therapy that simultaneously targeted both the entry pathways would be advantageous over therapies targeting the pathways individually. To this end, we developed a mathematical model of SARS-CoV-2 dynamics under treatment with a TMPRSS2 inhibitor, a Cathepsin B/L (or endosomal pathway) inhibitor, or both. We explicitly accounted for the dependence of viral entry efficiency via the two pathways on the expression levels of the two proteases and the effects of the drugs in suppressing the respective efficiencies in a dose-dependent manner. Our model predicted that targeting the two pathways simultaneously was likely not only to be efficacious but also synergistic.

Synergy between drugs implies that their combined effect is more than their individual effects added together [40–43]. Synergistic drug combinations are preferable because they allow the realization of the desired efficacy with lower net drug exposure, thus reducing toxicity. Conversely, at dosages limited by toxicities, synergistic drug combinations achieve higher efficacies than their non-synergistic counterparts. Synergy is thought to arise typically as the result of downstream interactions between the steps/pathways targeted by the drugs [40–43]. The synergy we predict here is thus surprising because the two entry pathways targeted are independent. Using our modelling and analysis, we show how this synergy arises from effects both at the single cell level as well as at the cell population level. We show further that the synergy is evident, although unrecognized, in available *in vitro* experiments with SARS-CoV-2 and SARS-CoV, providing evidence in strong support of our model predictions.

While the availability of the two pathways for entry is well recognized, their relative usage has not been established except in cell types where one of the pathways dominates overwhelmingly [21,28]. Our formalism allowed us to analyse available *in vitro* data and quantify the relative usage of the two pathways in different cell types. We estimated thus that in Vero cells expressing TMPRSS2, infection proceeded ~65% of the time via the TMPRSS2 pathway and ~35% via the Cathepsin B/L pathway. The usage was similar with SARS-CoV. The usage, however varied widely across cell lines, suggesting, given the potentially broad tissue tropism of SARS-CoV-2, extending beyond the lungs [21,44], that targeting both pathways may be important and synergistic *in vivo*. Together, our findings could help optimize combination therapies targeting TMPRSS2 and Cathepsin B/L for preventing SARS-CoV-2 entry.

## Results

### Overview of the mathematical model

**Fig 1:**
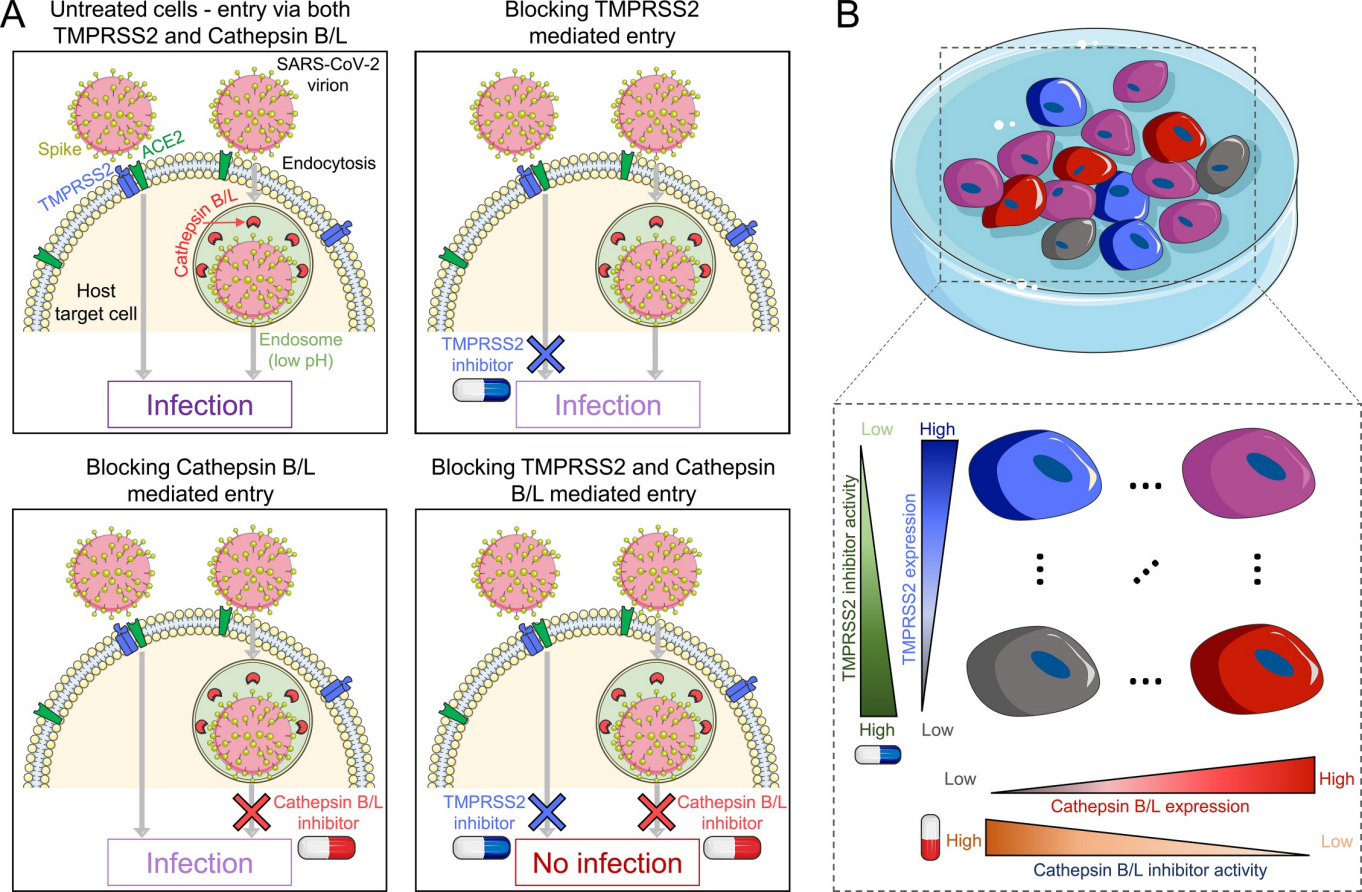
Schematic of the mathematical model incorporating the two independent pathways of SARS-CoV-2 entry into target cells. **(A)** After the SARS-CoV-2 virion attaches to the host surface receptor ACE2, S protein activation occurs by the host proteases TMPRSS2 (transmembrane) or Cathepsin B/L (endosomal) thus yielding two independent entry pathways. Blocking both pathways is essential for preventing infection of the cell expressing both proteases. **(B)** TMPRSS2 and Cathepsin B/L expression levels vary across cells. The entry efficiency through a pathway increases and the efficacy of the inhibitor at a given concentration decreases with the expression level of the corresponding protease.

We considered *in vitro* experiments where a population of target cells is exposed in the presence or absence of protease inhibitors to virions expressing the SARS-CoV-2 spike protein, S, and the extent of infection is measured. We constructed a mathematical model of the ensuing dynamical processes, focusing on virus entry and the role of the protease inhibitors (Fig 1). We assumed that cells could get infected following S protein activation by either TMPRSS2 or Cathepsin B/L. The efficiency of virus entry via the two pathways depended on the expression levels of the respective proteases. We denoted as *S*_*t*_ the susceptibility of a target cell expressing *n*_*t*_ copies of TMPRSS2 to entry via the TMPRSS2 pathway. The susceptibility here was also the probability with which a virion could successfully enter the cell when TMPRSS2 was the sole limiting factor. Analogously, *S*_*c*_ was the susceptibility to entry via the Cathepsin B/L pathway of a cell expressing *n*_*c*_ copies of Cathepsin B/L. The overall susceptibility, which we denoted *S*_*tc*_, of a cell expressing *n*_*t*_ and *n*_*c*_ copies of the two proteases, respectively, was then *S*_*tc*_ = *S*_*t*_+*S*_*c*_−*S*_*t*_*S*_*c*_, indicating the independence of the two pathways. We let *S*_*t*_ and *S*_*c*_ increase with *n*_*t*_ and *n*_*c*_, respectively, following distinct Hill functions (Methods). We assumed that drugs acted by suppressing the proteases in a dose-dependent manner. A TMPRSS2 inhibitor thus blocked a fraction of the *n*_*t*_ TMPRSS2 molecules, reducing *S*_*t*_. A Cathepsin B/L inhibitor similarly lowered *S*_*c*_. Furthermore, we assumed that a TMPRSS2 inhibitor left *S*_*c*_ unaffected, so that entry could proceed uninhibited via the Cathepsin B/L pathway even when a TMPRSS2 inhibitor was present in excess. Similarly, a Cathepsin B/L inhibitor would let entry occur uninhibited via the TMPRSS2 pathway. The drugs too thus acted independently. Only when both the drugs were used could a cell be fully protected (Fig 1A). We recognized that the cells in culture exhibited a distribution of the expression levels of TMPRSS2 and Cathepsin B/L and thus had a distribution of susceptibilities to infection via the two pathways (Fig 1B). The effects of the drugs too, thus, exhibited cell-to-cell variability. With this description, we formulated dynamical equations to predict how the population of cells in culture would get infected and how the drugs would suppress the infection (Methods). The model was solved using parameter values listed in S1 Table. Based on the model, we derived analytical expressions of the synergy between the drugs and elucidated its origins (S1 Text and S2 Text). Finally, we applied the model to analyse *in vitro* data and estimated the relative usage of the two pathways (Methods).

### The activity of TMPRSS2 and Cathepsin B/L inhibitors depends on the relative usage of the respective pathways

**Fig 2:**
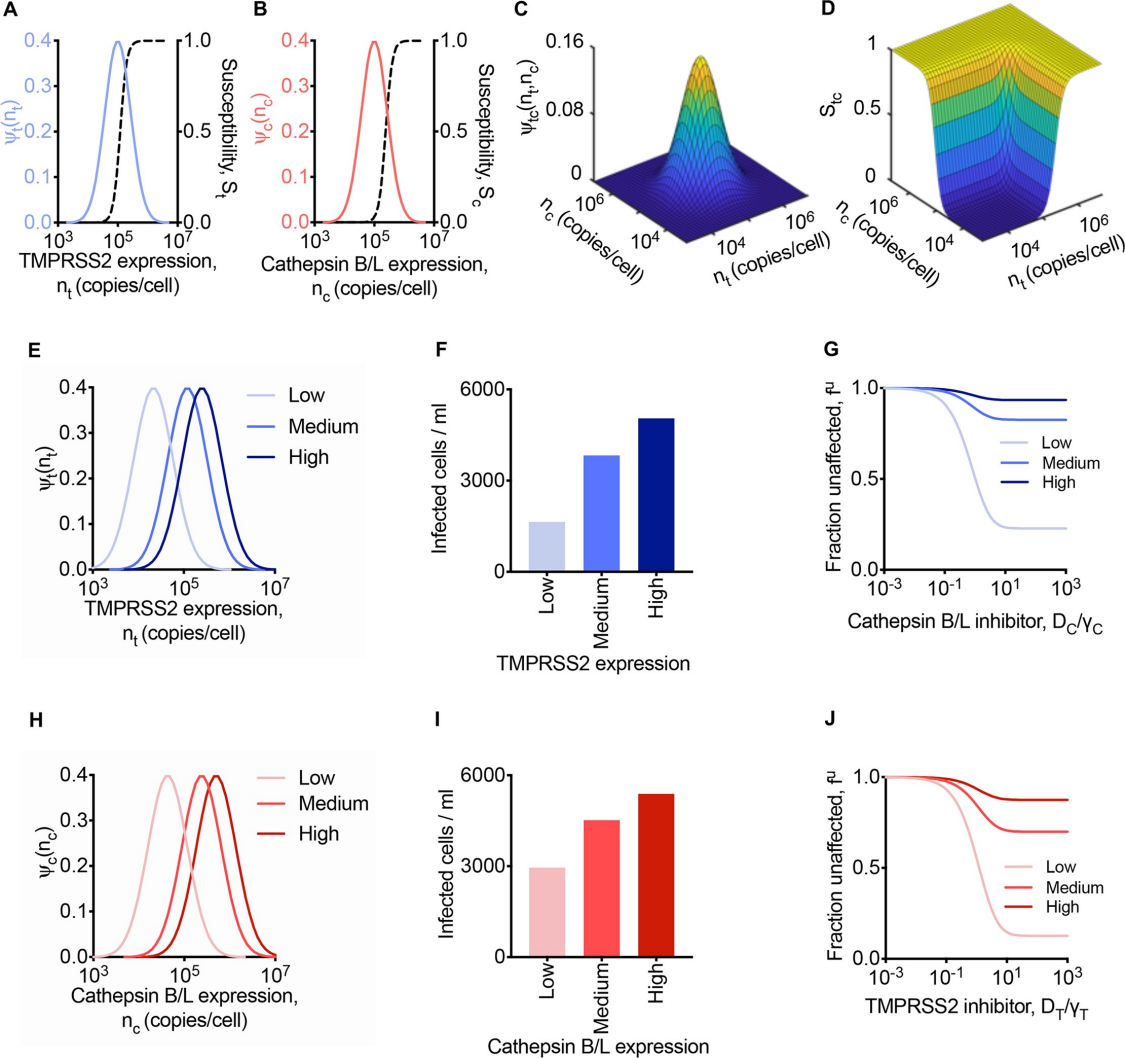
Predictions of SARS-CoV-2 entry into cells with heterogenous protease expression levels. **(A, B)** The log-normal distribution of TMPRSS2 (A) and Cathepsin B/L (B) across cells. Dependence of the susceptibility of infection through the TMPRSS2 pathway on the TMPRSS2 expression level, *n*_*t*_, (A) and through the Cathepsin B/L pathway on the Cathepsin B/L expression level, *n*_*c*_ (B). **(C)** The joint distribution of cells expressing both TMPRSS2 and Cathepsin B/L. **(D)** Dependence of susceptibility of infection on both TMPRSS2 and Cathepsin B/L expression levels. In (A)-(D), $\bar{n_{t}}$ = 11.5 and $\bar{n_{c}}$ = 11.5. **(E)** The log-normal distribution of TMPRSS2 across cells with low ($\bar{n_{t}}$ = 10), medium ($\bar{n_{t}}$ = 11.7) and high ($\bar{n_{t}}$ = 12.4) mean TMPRSS2 expression levels. **(F, G)** Infected cells in the absence of inhibitors (F) and fraction of infection events uninhibited by different concentrations of a Cathepsin B/L inhibitor (G) at different mean TMPRSS2 expression levels and fixed mean Cathepsin B/L expression ($\bar{n_{c}}$ = 11.5). **(H)** The log-normal distribution of Cathepsin B/L across cells with low ($\bar{n_{c}}$ = 10.7), medium ($\bar{n_{c}}$ = 12.4) and high ($\bar{n_{c}}$ = 13.1) mean Cathepsin B/L expression levels. **(I, J)** Infected cells in the absence of inhibitors (I) and fraction of infection events uninhibited by different concentrations of TMPRSS2 inhibitor (J) at different mean Cathepsin B/L expression levels and fixed mean TMPRSS2 expression ($\bar{n_{t}}$ = 11.5). In (F), (G), (I), and (J), the cumulative level of infection was computed at day 1 post infection, and the drug concentrations were normalised by the respective *γ* values (Methods). The other parameters and initial conditions are listed in S1 Table.

We considered cells expressing TMPRSS2 and Cathepsin B/L following independent, log-normal distributions (Fig 2A and 2B). The susceptibilities, *S*_*t*_ and *S*_*c*_, of the cells to entry via the two pathways were modelled as rising sigmoidally from zero at low expression levels to 1 at high expression levels of the respective proteases (Fig 2A and 2B). The joint distribution of the expression levels of TMPRSS2 and Cathepsin B/L on cells was given by the product of the individual distributions (Fig 2C). The overall susceptibility, *S*_*tc*_, was predicted to be small when both proteases were at low expression levels, rose with the expression levels, and reached 1 when either expression level was high (Fig 2D).

With this description of the susceptibility of cells to entry, we examined first the influence of TMPRSS2 expression levels on virus entry. We considered scenarios where the mean TMPRSS2 level was low, medium, or high, which corresponded to *S*_*t*_ at the mean expression levels of 0.001, 0.5, and 0.95, respectively (Fig 2E). Note that TMPRSS2 levels can vary substantially across tissues in the body, with the prostate, for instance, displaying significantly higher expression levels than the lung, trachea, or salivary glands [45–48]. Different cell lines too express widely varying TMPRSS2 levels [49]. For the distributions chosen, our model predicted that the total population of cells infected increased with TMPRSS2 expression, going from ~1640 cells/ml at low to ~5050 cells/ml infected at high mean TMPRSS2 expression level (out of 10^5^ initial target cells/ml) at 1 d post-infection (Fig 2F). The initial distribution of Cathepsin B/L expression (Fig 2B) was unaltered in these calculations. The susceptibility, *S*_*c*_, through the Cathepsin B/L pathway at the mean Cathepsin B/L expression level was 0.029. Thus, the utilization of the TMPRSS2 pathway (*S*_*t*_/*S*_*c*_) was, on average, ~30-fold lower, 17.1-fold higher, and 32.5-fold higher than the Cathepsin B/L pathway for the low, medium and high TMPRSS2 scenarios, respectively, indicating that as the TMPRSS2 level increased, it became increasingly preferred as the entry pathway in our predictions.

We reasoned that as the preference for the TMPRSS2 pathway increased, it would compromise the activity of a Cathepsin B/L inhibitor. We examined this by performing the calculations above but in the presence of a Cathepsin B/L inhibitor. Mimicking experiments [21], we calculated the fraction of infection events ‘unaffected’ by the Cathepsin B/L inhibitor, which we denoted *f*^*u*^(*D*_*C*_) (Fig 2G). Thus, *f*^*u*^(*D*_*C*_) = 1 would imply that the drug prevented no entry events, whereas *f*^*u*^(*D*_*C*_) = 0 would imply that the drug protected all cells that would have been infected otherwise. Our model predicted that as the inhibitor level increased, *f*^*u*^(*D*_*C*_) decreased, indicating increased drug activity (Fig 2G). *f*^*u*^(*D*_*C*_), however, did not reach 0 even at arbitrarily high drug concentrations. Instead, it plateaued at values that depended on the mean TMPRSS2 expression level. When the latter expression level was low, *f*^*u*^(*D*_*C*_) plateaued at ~0.23, indicating strong protection, whereas when the expression level was high, *f*^*u*^(*D*_*C*_) was ~0.93, implying little protection. Thus, the ‘apparent’ efficacy of the Cathepsin B/L inhibitor was dependent on TMPRSS2 expression. The inhibitor was efficacious when the predominant pathway was via Cathepsin B/L, which here happened when TMPRSS2 expression was low. When the latter expression was high, Cathepsin B/L was utilized negligibly; blocking it would thus have a minimal effect on virus entry, rendering the inhibitor poorly efficacious.

In the same way, we predicted that the apparent activity of TMPRSS2 inhibitors would depend on the expression level of Cathepsin B/L. When we used low, medium or high mean expression levels of Cathepsin B/L in our calculations (Fig 2H), with the TMPRSS2 expression level unaltered (Fig 2A), we found that the extent of infection increased in the absence of drugs from ~2960 cells/ml at low to ~5390 cells/ml at high Cathepsin B/L expression (Fig 2I). With a TMPRSS2 inhibitor, the extent of infection decreased, but the fraction unaffected, now denoted *f*^*u*^(*D*_*T*_), plateaued at values that increased with the mean Cathepsin B/L expression level– *f*^*u*^(*D*_*T*_)~0.13 at low and *f*^*u*^(*D*_*T*_)~0.88 at high expression levels–indicating again that the apparent activity of the TMPRSS2 inhibitor decreased as the Cathepsin B/L pathway became increasingly preferred (Fig 2J).

These predictions were consistent with several experiments showing that high expression levels of TMPRSS2 led to poor efficacies of Cathepsin B/L inhibitors [21,27–29]. For instance, when 293T cells expressing human ACE2 but not TMPRSS2 were infected with SARS-CoV in the presence of 25 mM NH_4_Cl, which prevents endosomal acidification and hence blocks Cathepsin B/L, the extent of infection reduced ~40-fold over that in the absence of NH_4_Cl [29]. When the same cells were engineered to express TMPRSS2, the reduction was just ~4-fold, marking a drastic loss of the apparent activity of NH_4_Cl. Similar effects were observed with other endosomal pathway inhibitors including E-64d, MDL28170, EST, and bafilomycin [21,27–29]. Importantly, recent experiments observed this effect with SARS-CoV-2 [21]. When 293T cells expressing ACE2 were infected with SARS-CoV-2 pseudotyped viruses, treatment with E-64d reduced the infection by 90%, whereas when the cells additionally expressed TMPRSS2, the effect of E-64d nearly vanished [21].

The predictions were also consistent with experiments showing high activity of TMPRSS2 inhibitors when the expression level of TMPRSS2 was high [21,28]. For instance, with HeLa cells expressing ACE2, the effect of camostat mesylate was negligible in inhibiting SARS-CoV infection compared to its effect on the same cells engineered to overexpress TMPRSS2 [28]. With the latter cells, camostat mesylate prevented ~65% of infections at the highest doses, consistent with the plateau predicted by our model (Fig 2G and 2J). The implication was that the remaining infection occurred through the Cathepsin B/L pathway. In the same way, with Vero cells, which hardly express TMPRSS2, camostat mesylate had little effect in reducing SARS-CoV-2 infection, whereas E-64d, which blocked Cathepsin B/L reduced nearly 100% of the infections [21]. At the same time, in Vero cells engineered to overexpress TMPRSS2, with the same dosages of the drugs, E-64d could only block ~25% of the infections, whereas camostat mesylate blocked nearly ~65% of the infections, indicative again of the relative utilization of the pathways in these cell lines. Similar results were observed also with SARS-CoV infection [21].

These findings suggest caution when interpreting assays measuring the efficacies of drugs targeting host proteases. A drug may block its target protease potently but may still appear poorly efficacious at preventing viral entry because of the activity of the other protease. More importantly, the findings suggest that with cells expressing significant levels of both TMPRSS2 and Cathepsin B/L, the use of either a TMPRSS2 inhibitor or a Cathepsin B/L inhibitor alone would be insufficient to block entry completely. The inhibitors would have to be used together to completely block entry. We therefore examined next our model predictions when the inhibitors were used together.

### The combined use of TMPRSS2 and Cathepsin B/L inhibitors can fully block entry

**Fig 3:**
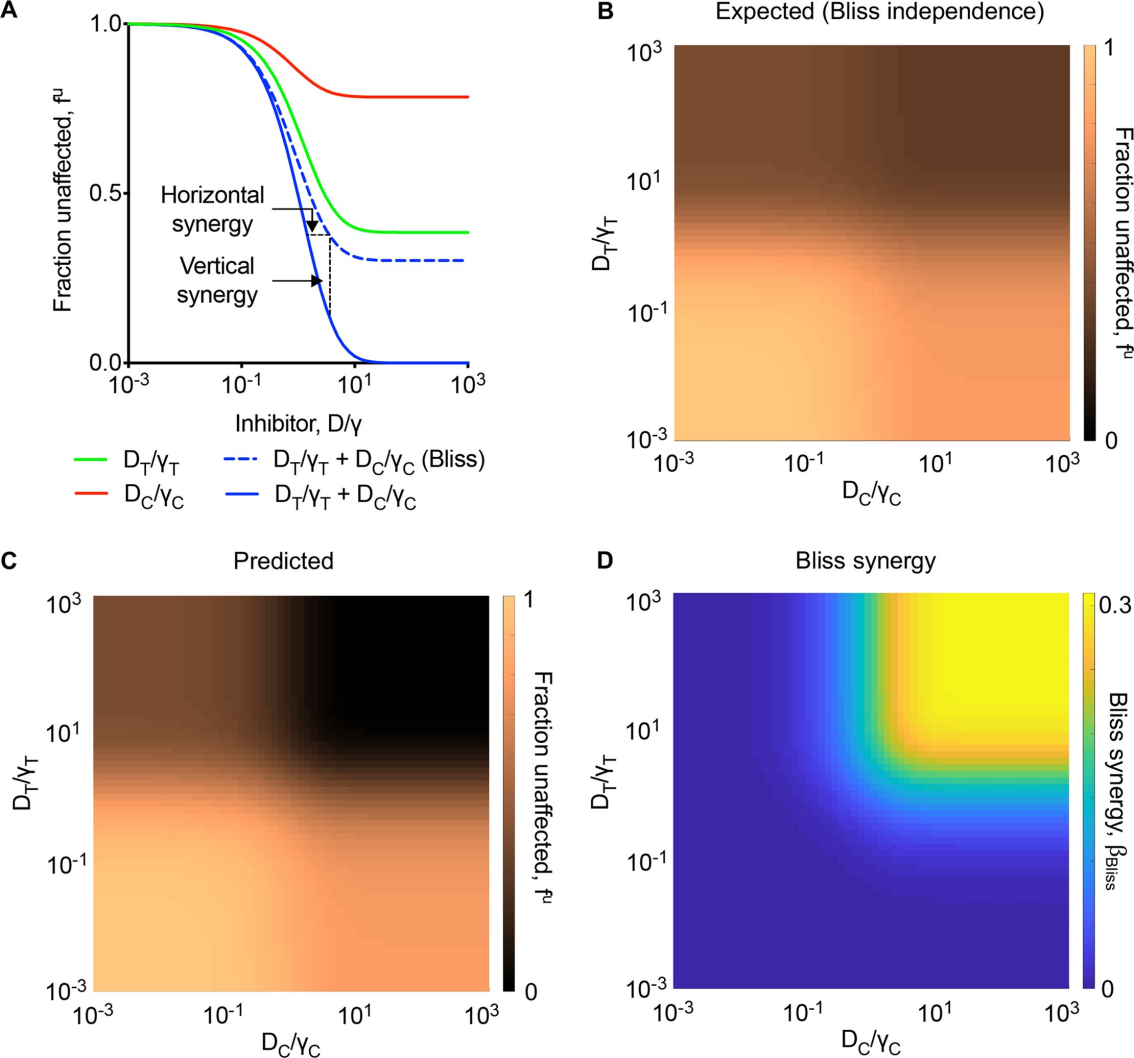
Predictions of the effect of combination treatment targeting both TMPRSS2 and Cathepsin B/L pathways. **(A)** The fraction of infection events unaffected by Cathepsin B/L inhibitor, TMPRSS2 inhibitor or both for different drug concentrations. The extent of predicted horizontal and vertical or Bliss synergy (see text) are marked. **(B, C)** The expected effect of the combination from Bliss independence (B) and predicted combination effect (C) over varying drug concentrations. **(D)** The predicted Bliss synergy over a range of drug concentrations. The parameters and initial conditions are listed in S1 Table.

To elucidate the effect of combination therapy, we predicted the effects of the drugs alone and in combination in blocking virus entry. Because the cells expressed both proteases, the drugs individually could not block entry fully. For the parameters chosen, the effect of the Cathepsin B/L inhibitor plateaued at *f*^*u*^(*D*_*C*_)~0.78 and that of the TMPRSS2 inhibitor at *f*^*u*^(*D*_*T*_)~0.39 when used alone (Fig 3A). When the two were combined, our model predicted 100% block of entry; the fraction unaffected, now denoted *f*^*u*^(*D*_*T*_,*D*_*C*_), plateaued at 0 (Fig 3A).

This prediction was consistent, again, with several *in vitro* studies showing that full blockade of entry required the combined use of TMPRSS2 and Cathepsin B/L inhibitors [21,28]. With HeLa cells expressing ACE2 and TMPRSS2, EST alone blocked ~34% of SARS-CoV infection, camostat mesylate blocked ~58%, and the two together blocked ~100% of the infections [28]. In Vero cells expressing TMPRSS2, the corresponding numbers were ~20% for E-64d, ~47% for camostat mesylate, and ~98% for both with SARS-CoV infection [21]. Importantly, the latter numbers were ~21%, ~57%, and ~92%, respectively, for SARS-CoV-2 infection [21].

### TMPRSS2 and Cathepsin B/L inhibitors synergize in blocking virus entry

The above predictions indicated strong synergy between drugs blocking the two entry pathways (Fig 3A). Because the pathways are independent, the combined effect of the drugs is expected to follow Bliss independence, where the fraction of events unaffected by the combination is the product of the fractions unaffected when the drugs are used alone [40,43,50]. In other words, Bliss independence would imply $f_{Bliss}^{u}(D_{T},D_{C})=f^{u}(D_{T})\times f^{u}(D_{C})$. If *f*^*u*^(*D*_*T*_,*D*_*C*_) is smaller than $f_{Bliss}^{u}(D_{T},D_{C})$, the drugs exhibit synergy; *i*.*e*., they block more infections together than expected from their individual effects put together. With the above parameter values, where *f*^*u*^(*D*_*T*_)~0.39 and *f*^*u*^(*D*_*C*_)~0.78, Bliss independence would yield $f_{Bliss}^{u}(D_{T},D_{C})∼0.3$. The effect predicted by our model, however, was *f*^*u*^(*D*_*T*_,*D*_*C*_) = 0, implying strong synergy (Fig 3A).

The synergy can be visualized in two ways [40]: horizontal and vertical synergy (Fig 3A). Horizontal synergy refers to the decrease in drug levels from that assuming independent action that is required to produce the same effect as that achieved by Bliss independence. Vertical synergy refers to the gain in efficacy at fixed drug levels over that assuming Bliss independence. The two are equivalent ways of quantifying synergy. The latter has been termed Bliss synergy and is more widely used [43,50–52]. Here, we employed the latter measure throughout and denoted it as $\beta_{Bliss}=f_{Bliss}^{u}(D_{T},D_{C})-f^{u}(D_{T},D_{C})$. As the extent of synergy increases, *β*_*Bliss*_ increases. Note that 0≤*β*_*Bliss*_≤1 here. (Negative values of *β*_*Bliss*_ indicate antagonistic interactions between the drugs.)

To assess the extent of synergy that could be realized, we performed the above calculations over wide ranges of drug levels. We predicted that the combined efficacy expected assuming Bliss independence was small ($f_{Bliss}^{u}(D_{T},D_{C})∼1$) when the drug levels were small and rose to the maximum estimated above ($f_{Bliss}^{u}(D_{T},D_{C})∼0.3$) at saturating drug levels (Fig 3B). In striking contrast, the predicted efficacy of the combination was much higher at the saturating drug levels (*f*^*u*^(*D*_*T*_,*D*_*C*_)~0) (Fig 3C). Correspondingly, *β*_*Bliss*_~0.3, quantifying the maximum extent of synergy realized for the parameters chosen (Fig 3D). These predictions were robust to parameter variations (S1 and S2 Figs).

The predictions were intriguing because the pathways are independent, and the combination should have accordingly followed Bliss independence. We examined next the possible origins of the synergy in our model.

### Origin of synergy at the single cell level

Synergy could arise from the effects of the drugs at the single cell level [51,53,54] or at the cell population level [53,55–57]. We sought to examine first whether synergy could arise in our calculations from effects at the single cell level. For this, we considered cells that had the same TMPRSS2 and Cathepsin B/L levels, eliminating heterogeneity in the cell population. Any synergy would then have to arise from effects at the single cell level. We repeated our calculations above (Figs 2 and 3) with this homogeneous cell population.

**Fig 4:**
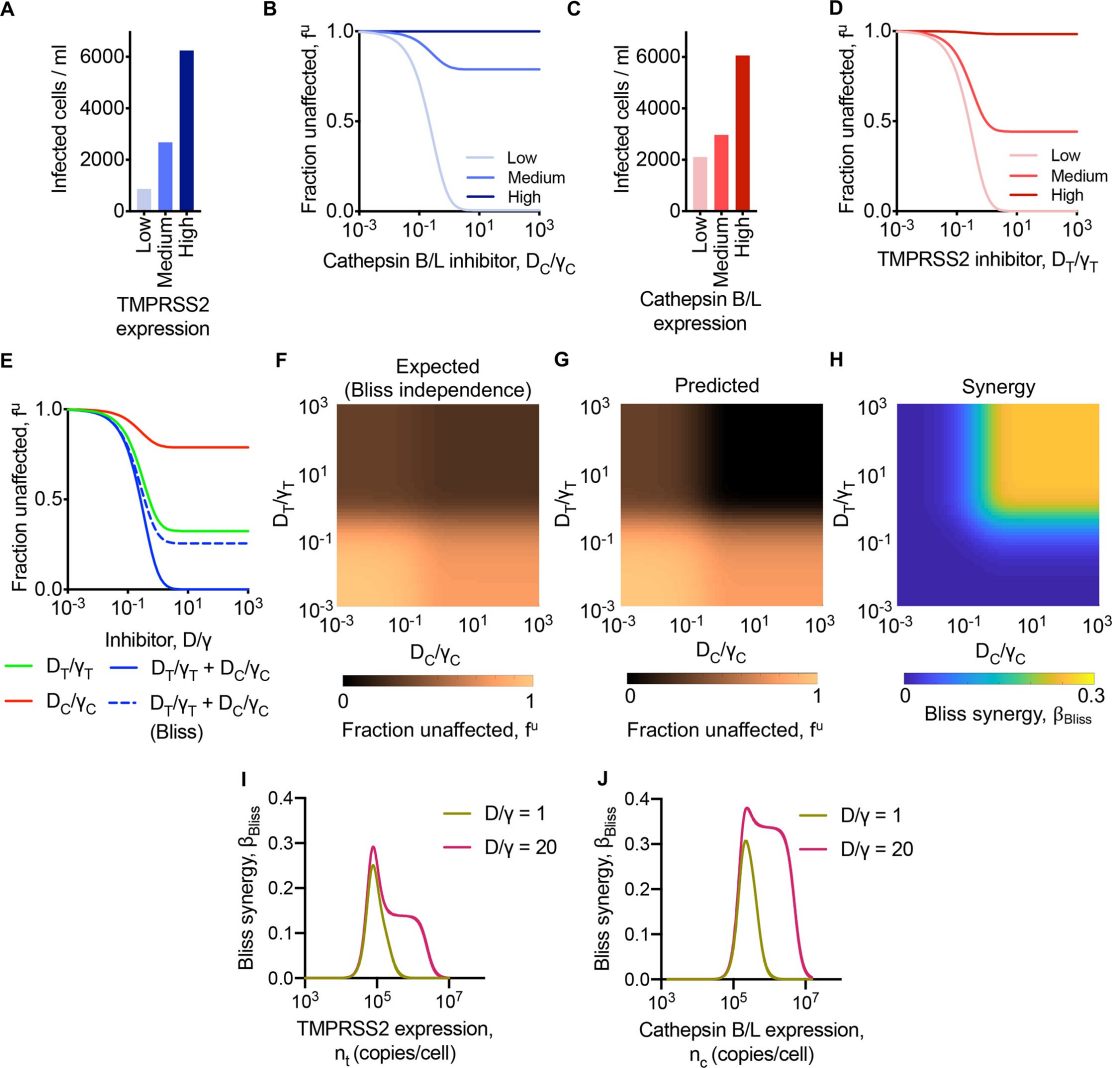
Predictions of SARS-CoV-2 entry into cells with homogenous protease expression levels. **(A, B)** Cells infected in the absence of inhibitors (A) and fraction of infection events uninhibited by different concentrations of Cathepsin B/L inhibitor (B) in a population of cells expressing low (*n*_*t*_ = 20,000 copies/cell), medium (*n*_*t*_ = 100,000 copies/cell) and high (*n*_*t*_ = 500,000 copies/cell) TMPRSS2 levels and fixed (*n*_*c*_ = 150,000 copies/cell) Cathepsin B/L level. **(C, D)** Cells infected in the absence of inhibitors (C) and fraction of infection events uninhibited by different concentrations of TMPRSS2 inhibitor (D) in a population of cells expressing low (*n*_*c*_ = 20,000 copies/cell), medium (*n*_*c*_ = 170,000 copies/cell) and high (*n*_*c*_ = 500,000 copies/cell) Cathepsin B/L levels and fixed (*n*_*t*_ = 100,000 copies/cell) TMPRSS2 level. **(E)** The fraction of infection events unaffected by Cathepsin B/L inhibitor, TMPRSS2 inhibitor or both for different drug concentrations in cell population with *n*_*t*_ = 100,000 copies/cell and *n*_*c*_ = 150,000 copies/cell. **(F, G)** The expected combination effect from Bliss independence (F) and predicted combination effect (G) over a varying levels of drug concentrations. **(H)** The predicted Bliss synergy over a range of drug concentrations. **(I, J)** The predicted Bliss synergy for varying TMPRSS2 expression and fixed (*n*_*c*_ = 150,000 copies/cell) Cathepsin B/L expression and for varying Cathepsin B/L expression and fixed (*n*_*t*_ = 100,000 copies/cell) TMPRSS2 expression at two different drug concentrations. The drug concentrations were normalised by the respective *γ* values. The parameters and initial conditions are listed in S1 Table.

We found that for fixed Cathepsin B/L levels, as the expression level of TMPRSS2 increased from low to high, the number of cells infected increased from ~870/ml to ~6240/ml in the absence of drugs (Fig 4A). With a Cathepsin B/L inhibitor, *f*^*u*^(*D*_*C*_) decreased and reached a plateau (Fig 4B). The plateau was lower, indicating greater drug efficacy, as the TMPRSS2 level decreased, consistent with the observations above of the drug efficacy increasing as the relative usage of the corresponding pathway increased. Analogous predictions resulted upon increasing Cathepsin B/L expression levels at a fixed TMPRSS2 level (Fig 4C) and in the presence of increasing concentrations of a TMPRSS2 inhibitor (Fig 4D).

For our calculations with both the drugs present simultaneously, we chose protease expression levels such that the individual drug effects plateaued at *f*^*u*^(*D*_*T*_)~0.32 and *f*^*u*^(*D*_*C*_)~0.79, respectively (Fig 4E). In the absence of synergy, the expected combined effect would plateau at $f_{Bliss}^{u}(D_{T},D_{C})∼0.26$. Our model predicted instead that the combined effect plateaued at *f*^*u*^(*D*_*T*_,*D*_*C*_)~0, indicating strong synergy at the single cell level (Fig 4E). Specifically, the extent of Bliss synergy, denoted $\beta_{Bliss}^{cell}$ to emphasize its origin, was $\beta_{Bliss}^{cell}∼0.26$. Spanning a wide range of drug concentrations, we found that $f_{Bliss}^{u}(D_{T},D_{C})$ decreased from ~1 at low drug levels and plateaued at ~0.26 at high drug levels (Fig 4F), whereas *f*^*u*^(*D*_*T*_,*D*_*C*_) decreased further and reached zero at high levels (Fig 4G). The corresponding synergy was also low at low drug levels and rose to $\beta_{Bliss}^{cell}∼0.26$ at high drug levels, reiterating the existence of synergy at the single cell level and quantifying its extent (Fig 4H).

The above calculations were for fixed TMPRSS2 and Cathepsin B/L levels. To ascertain the robustness of the results, we repeated the calculations with different levels of either TMPRSS2 (Fig 4I) or Cathepsin B/L (Fig 4J), while keeping all the other parameters fixed. We found that the synergy had a non-monotonic dependence on the expression levels. As the TMPRSS2 level increased, for instance, $\beta_{Bliss}^{cell}$ rose from zero, attained a peak that was dependent on the drug level, and then declined again to zero (Fig 4I). We explain this effect by considering again the relative preferences for the two entry pathways. When the TMPRSS2 level was low, its contribution to entry was low, so that only Cathepsin B/L inhibitors would display significant efficacy. The effect of the TMPRSS2 inhibitor was imperceptible. No synergy could arise. Conversely, when the TMPRSS2 levels were high, Cathepsin B/L inhibitors had hardly any effect. Synergy was again not possible. At intermediate expression levels, synergy could occur and was maximized. Thus, synergy was high when the relative usage of the two pathways were comparable and vanished when one of the two pathways dominated. With increasing drug levels, this effect was amplified because the drugs blocked their respective pathways more effectively, leading to increased overall synergy at intermediate expression levels. Analogous effects were evident with varying Cathepsin B/L expression levels (Fig 4J).

These trends also emerged in analytical expressions we derived for synergy in single round infection assays [21,28], providing a deeper understanding of the synergy at the single cell level (S1 Text). Indeed, predictions using the analytical expressions were in close agreement with those of our model above (S3 Fig).

We examined next whether synergy could also arise from effects at the cell population level.

### Origin of synergy at the cell population level

**Fig 5:**
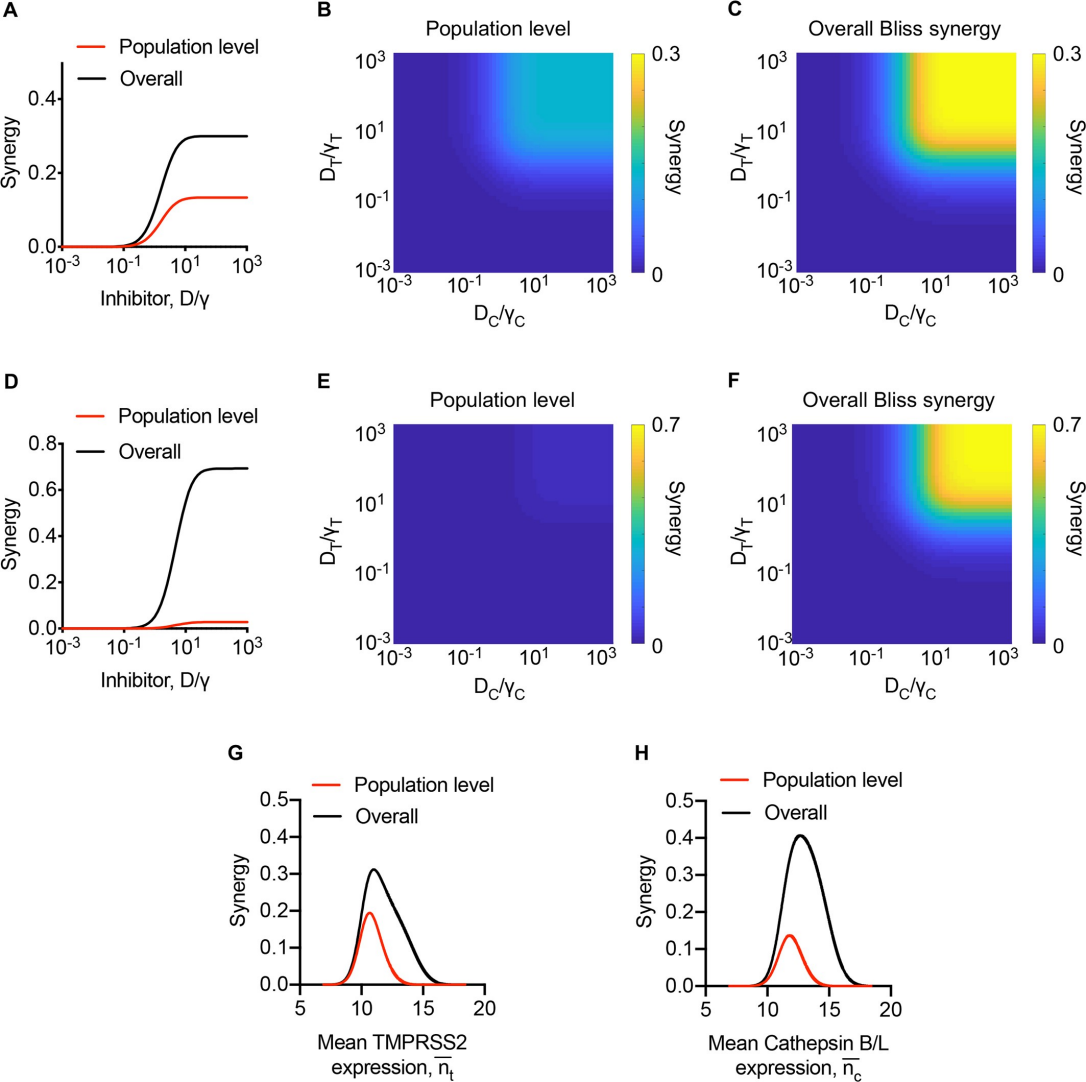
Predictions of population level synergy. **(A-F)** The predicted population level and overall synergy over a range of drug concentrations (see text for details). **(G, H)** The predicted overall and population level synergy for varying mean TMPRSS2 expression and fixed mean ($\bar{n_{c}}$ = 11.5) Cathepsin B/L expression (G) and for varying mean Cathepsin B/L expression and fixed mean ($\bar{n_{t}}$ = 11.5) TMPRSS2 expression at two different drug concentrations (H). The drug concentrations were normalised by the respective *γ* values. In A-C and G-H, $n_{t}^{50}$ = 1.2 x 10^5^ copies/cell and $n_{c}^{50}$ = 2.4 x 10^5^ copies/cell. In D-F, $n_{t}^{50}$ = 4 x 10^4^ copies/cell, $n_{c}^{50}$ = 4 x 10^4^ copies/cell. In G-H, *D*_*T*_/*γ*_*T*_ = *D*_*C*_/*γ*_*C*_ = 10. The parameters are listed in S1 Table.

Synergy at the cell population level can arise when heterogeneity exists in the response of cells to the drugs involved [53,55–57]. Here, such heterogeneity could arise from the distributions of the expression levels of the two proteases across cells. The synergy emerging from such heterogeneity is evident in the extreme scenario we conceived where cells in culture either expressed TMPRSS2 or Cathepsin B/L but not both and for which we were able to derive analytical expressions of the synergy (S2 Text). To assess the extent of synergy arising from the cell population level in a more realistic scenario, we performed calculations akin to those in Figs 2 and 3 above (Methods). We predicted that when the overall synergy, *β*_*Bliss*_, reached a plateau at ~0.3, the cell population level synergy, $\beta_{Bliss}^{popn}$, was ~0.13, amounting to a contribution of nearly 43% to the total synergy (Fig 5A). By varying drug levels over wide ranges, we predicted that the synergy would be low at low drug levels, and increase monotonically as drug levels rise (Fig 5B). The single cell level synergy displayed similar trends, leading to the overall synergy also rising monotonically with drug levels and eventually saturating at ~0.3 (Fig 5C). We recognized from our analysis of the extreme scenario above that $\beta_{Bliss}^{popn}\leq 1/4$ (S2 Text), whereas $\beta_{Bliss}^{cell}$ can rise to 1 (S1 Text). Thus, depending on the overall synergy, the contribution from the cell population level may vary. Whereas it was ~43% above, it could be much lower if $\beta_{Bliss}^{popn}$ were smaller and/or $\beta_{Bliss}^{cell}$ were larger. To illustrate this, we performed calculations with parameter settings that yielded a much higher overall synergy as well as a lower cell population level synergy. Here, *β*_*Bliss*_ saturated at 0.69, whereas $\beta_{Bliss}^{popn}$ saturated at 0.03, resulting in a far smaller contribution, ~4.4%, from the latter (Fig 5D, 5E and 5F). Finally, we note that $\beta_{Bliss}^{popn}$ too displayed non-monotonic dependencies on the expression levels of the proteases (Fig 5G and 5H). These trends mimic those with the single cell level synergy (Fig 4I and 4J) and are understood from the analytical expressions we derived (S2 Text).

In summary, it followed from these model predictions that contributions could arise to the synergy from effects of the drugs at the single cell as well as cell population levels. We examined next whether the synergy we predicted was evident in available *in vitro* data on SARS-CoV-2 and SARS-CoV infection.

### TMPRSS2 and Cathepsin B/L inhibitors display synergy *in vitro*

We considered data from 4 independent experiments, 1 with SARS-CoV-2 and 3 with SARS-CoV, performed across 2 different cell lines, using camostat mesylate as the TMPRSS2 inhibitor and 3 different drugs as Cathepsin B/L inhibitors, in two independent studies that have examined the combined effect of blocking TMPRSS2 and Cathepsin B/L [21,28]. The studies exposed cells to pseudotyped viruses in the absence of drugs or in the presence of either or both drugs and reported the fraction of infection events unaffected by the drugs, namely, *f*^*u*^(*D*_*T*_), *f*^*u*^(*D*_*C*_), and *f*^*u*^(*D*_*T*_,*D*_*C*_). Using the latter data, we estimated the extent of Bliss synergy, *β*_*Bliss*_ = *f*^*u*^(*D*_*T*_)×*f*^*u*^(*D*_*C*_)−*f*^*u*^(*D*_*T*_,*D*_*C*_), between the drugs (Methods). We found that these experiments displayed synergy between TMPRSS2 and Cathepsin B/L inhibitors, in agreement with our model predictions (Table 1).

**Table 1:** Experimental evidence of Bliss synergy. We computed the extent of synergy from reported data on the effects of the individual drugs and the combination. The difference between the expected effect of the combination in terms of the fraction unaffected (*f*^*u*^) based on Bliss independence and the effect observed yielded the extent of synergy. Infection was by viruses pseudotyped with either SARS-CoV-2 S (denoted SARS-2-S) or SARS-CoV S (denoted SARS-S) protein. The experimental observations (*f*^*u*^(*D*_*T*_), *f*^*u*^(*D*_*C*_), and *f*^*u*^(*D*_*T*_,*D*_*C*_)) reported are reproduced for convenience and using which we computed the extent of synergy (*β*_*Bliss*_).

*Infection*	*Cell line*	*Drug combination*	*f*^*u*^(*D*_*T*_)	*f*^*u*^(*D*_*C*_)	*f*^*u*^(*D*_*T*_,*D*_*C*_)		*β*_*Bliss*_	*Ref*.
					*Expected*	*Observed*		
SARS-2-S	Vero-TMPRSS2	Camostat (*D*_*T*_) + E-64d (*D*_*C*_)	0.43	0.79	0.34	0.08	0.26	[21]
SARS-S	Vero-TMPRSS2	Camostat (*D*_*T*_) + E-64d (*D*_*C*_)	0.53	0.8	0.42	0.02	0.4	[21]
SARS-S	HeLa-ACE2-TMPRSS2	Camostat (*D*_*T*_) + EST (*D*_*C*_)	0.42	0.66	0.28	0.06	0.22	[28]
SARS-S	HeLa-ACE2-TMPRSS2	Camostat (*D*_*T*_) + Bafilomycin (*D*_*C*_)	0.42	0.6	0.25	0.08	0.17	[28]

**Fig 6:**
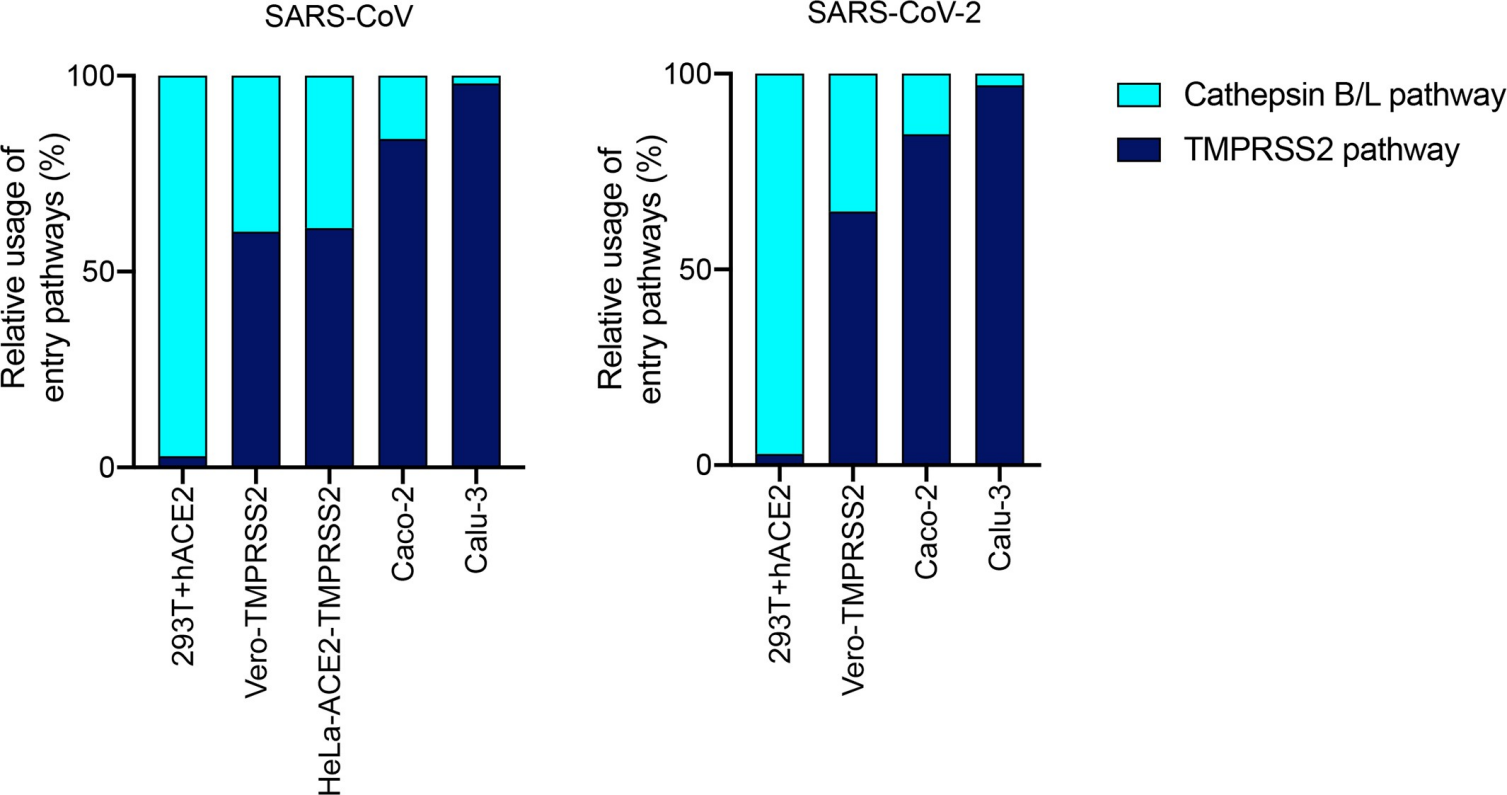
Relative usage of TMPRSS2 and Cathepsin B/L pathways during SARS-CoV and SARS-CoV-2 entry. The fraction of infection events unaffected either by TMPRSS2 or Cathepsin B/L inhibitors in excess were used to estimate the relative entry pathway usage in different cells lines. Data and sources are in S2 Table. The analysis follows the formalism outlined in the Methods.

*Note that synergy is not expected in cell lines such as Calu-3 and Caco-2 where one pathway dominates (see Fig 6).

With SARS-CoV-2, we estimated *β*_*Bliss*_~0.26 between camostat mesylate and E-64d in Vero cells expressing TMPRSS2, suggesting that the combination protected an additional 26% of target cells from being infected relative to the additive effects of the two drugs at the same level of exposure. Strong synergy was also seen with SARS-CoV, with *β*_*Bliss*_~0.4 under the same conditions. The synergy extended to other drug combinations and cell lines. With camostat mesylate and EST, the latter a broad inhibitor of cysteine proteases including Cathepsin B/L, *β*_*Bliss*_~0.22 with SARS-CoV infection in HeLa cells expressing ACE2 and TMPRSS2. With camostat mesylate and bafilomycin, which prevents endosomal acidification and thus blocks Cathepsin B/L function, the corresponding estimate was *β*_*Bliss*_~0.17.

This evidence of the strong presence of synergy between TMPRSS2 and Cathepsin B/L inhibitors validated our model predictions. The synergy could be exploited in defining optimal drug combinations targeting host proteases required for SARS-CoV-2 entry, as has been suggested in other contexts [40,43,51,52,54]. For such optimization, knowledge of the nature of synergy, *i*.*e*., whether it originates from the single cell or the cell population level, is important. We considered this next.

### The relative usage of the entry pathways varies widely across cell types

From our calculations above, synergy at the single cell level would depend on the susceptibility of individual cells to entry via both pathways, whereas synergy at the cell population level would depend on the heterogeneity in the susceptibilities across cells. To assess these possibilities, we applied our model next to available *in vitro* data such as the above and estimated the relative usage of the two pathways for entry in different cell lines (Methods). The different cell lines could be representative of the different cell and tissue types that could be infected *in vivo*. If the different cell lines predominantly used either one or the other pathway, synergy would arise mostly from the cell population level. On the other hand, if all the cell lines used both pathways with similar relative pathway usage, synergy would arise mostly from the single cell level.

We collated *in vitro* data of SARS-CoV and SARS-CoV-2 pseudotyped virus infection of several different cell lines across studies [21,28] (S2 Table) and analysed it using our model. We found that the relative usage of the pathways was similar between SARS-CoV and SARS-CoV-2 but varied widely across cell types (Fig 6). For instance, with 293T cells expressing ACE2, entry was nearly exclusively via the Cathepsin B/L pathway. With Vero cells expressing TMPRSS2, entry of SARS-CoV-2 occurred ~65% of the time via the TMPRSS2 pathway and ~35% of the time through the Cathepsin B/L pathway. The corresponding figures were ~60% and ~40% for SARS-CoV. The latter were also similar to SARS-CoV entry into HeLa cells expressing ACE2 and TMPRSS2. With Caco cells, the relative usage of the TMPRSS2 pathway increased further to ~85% for both SARS-CoV and SARS-CoV-2 and culminated at nearly 100% usage of the TMPRSS2 pathway in Calu3 cells.

This spectrum of the relative usage of the pathways across cell types is striking and spans the range from exclusive usage of one pathway to the nearly even usage of both. If this spectrum were indicative of the usage *in vivo*, which remains to be identified, it would imply that combination therapy targeting the two pathways would benefit from synergy arising from both the single cell level and the cell population level.

## Discussion

The unprecedented scale of the COVID-19 pandemic has spurred urgent efforts to develop drugs and vaccines against SARS-CoV-2. Targeting host proteases required for SARS-CoV-2 entry into target cells appears a promising option and is under clinical investigation. Here, we identified unexpected synergy between drugs that target the serine protease TMPRSS2 and the cysteine proteases Cathepsin B/L, which offer alternative and independent pathways for SARS-CoV-2 entry. Using mathematical modelling of SARS-CoV-2 entry via the two pathways, we showed that the synergy is novel and arises from effects both at the single cell level and the cell population level. We found analysing several reported *in vitro* experimental observations that the synergy was evident not only with SARS-CoV-2 but also with the closely related and much more extensively studied SARS-CoV infection, which uses the same entry pathways, and was observed across drugs and cell lines, suggesting that the synergy is a robust phenomenon. Further, we estimated the relative usage of the two pathways and found it to vary widely across cell types, suggesting that synergy from the single cell and cell population levels is likely to arise *in vivo*, given the broad tissue tropism already identified for SARS-CoV-2 infection [21,44]. Drugs targeting TMRPSS2, namely, camostat mesylate and nafamostat mesylate, as well as drugs targeting the Cathepsin B/L pathway, such as hydroxychloroquine, are in clinical trials for SARS-CoV-2 treatment [3,17]. Exploiting the synergy may maximize the impact of such drugs and improve the chances of curing SARS-CoV-2 infection.

Synergy can be of particular importance in the use of repurposed drugs. Because repurposed drugs are designed primarily for other, typically unrelated targets, they may display suboptimal efficacies against their newly intended targets. Indeed, a recent analysis of SARS-CoV-2 dynamics in 13 patients treated with different repurposed drugs found that the drug efficacies were in the range of 20–70% and were far below the minimum of 80% that would be required for effective treatment after the onset of symptoms [58]. Increasing drug levels to achieve desired efficacies is likely to be limited by toxicities. Synergy here could be particularly helpful for it would allow the realization of the desired efficacies at drug concentrations below what would be required had the drugs acted independently. Thus, the synergy between TMPRSS2 inhibitors and Cathepsin B/L inhibitors we unravelled may improve their deployability against SARS-CoV-2.

The synergy we elucidated is unconventional and arises because two independent entry pathways are accessible to SARS-CoV-2. Classically, synergy is thought to arise between drugs when one of the drugs potentiates the other through interactions between their targets or downstream pathways [40,41,43,50,52,59]. No such interaction is evident here. The synergy here arises because blocking both pathways is necessary for preventing entry and can only be achieved when both the drugs are used. Such synergy has been recognized earlier in the context of biochemical pathways where the same pathway can be triggered by two upstream stimuli independently [40]. Drugs that block the two stimuli, seemingly independent targets, display synergy, in a manner similar to what we have elucidated here. In addition, synergy arises in our case from the variations in the expression levels of the two proteases across cells. Because of the variations, some cells predominantly use one entry pathway and others the other pathway. Thus, protecting the entire cell population is achieved efficaciously when both the drugs are used. Such cell population level synergy has been argued to explain the observed synergy between drugs targeting hepatitis C virus entry and those targeting other steps of the hepatitis C virus lifecycle including type I interferons [55,60]. The overall synergy seen here is thus a convolution of the synergy at the single cell level as well as that at the cell population level. To our knowledge, such multi-level synergy between drugs has not been recognized earlier.

The multi-level synergy implies that the use of the drugs in combination may address redundancies at multiple levels that sustain the infection. The redundancy at the level of protease usage for infecting individual cells is well recognized [21,28]. The combination would block both TMPRSS2 and Cathepsin B/L, addressing the redundancy and thereby protecting cells. Redundancy may also arise at the cell population level. The cell tropism of SARS-CoV-2 in humans is only beginning to be identified and is speculated to be wider than SARS-CoV [46]. The expression level of TMPRSS2 varies across tissues in humans and mice [45–47]. It is possible thus that in some tissues, with high TMPRSS2 expression, SARS-CoV-2 predominantly uses TMPRSS2 for entry, whereas in others, with low TMPRSS2 expression, it uses Cathepsin B/L. Indeed, we estimated the relative usage of the entry pathways and found it to vary widely across cell lines, possibly indicative of the heterogeneity that may be expected *in vivo*. Protecting all the target tissues is thus only possible with the combination. If the extent of these redundancies *in vivo*, which remains to be ascertained, is high, the extent of synergy and hence the efficacy of the combination would be high.

Recently, a trade-off between synergy and efficacy has been identified in a variety of synergistic drug combinations, including those used in HIV, hepatitis C and cancer treatments [52,54]. The trade-off implies that as the efficacy of a combination increases beyond a point, the synergy decreases, thereby limiting the synergy that can be realized given a desired efficacy. This trade-off originates from the classical drug interactions underlying synergy, where, for instance, one drug potentiates the other in blocking different steps of the same pathway [52]. If one of the drugs is used at such high efficacies that the pathway is nearly completely blocked, then little room is left for the other drug to act and no synergy is possible. This trade-off is not expected to constrain the combination of TMPRSS2 and Cathepsin B/L inhibitors because the two target independent pathways and not different steps in the same pathway. Indeed, our model predictions showed that the synergy would increase with efficacy, implying a gain in synergy with increasing drug concentrations. The synergy may be compromised, however, and become constrained by the trade-off if one of the drugs were to exhibit activity against both the pathways, as has been suggested with the drug TLCK [28].

Mathematical models have been developed earlier to describe receptor-dependent virus entry, with a focus on defining the entry requirements of viruses and quantifying targets of entry inhibitors and vaccines [55,61–70]. The models, however, have focussed typically on a single entry pathway, although multiple proteins may have been involved in mediating entry through the pathway. For instance, models of HIV-1 entry, which requires the binding of the HIV-1 envelope gp120 to the cell surface receptor CD4 and the chemokine receptor CCR5 (or CXCR4), have focused on estimating the number of gp120 trimers that must engage with CD4 and CCR5 for successful entry [62–64,67]. Similarly, models of hepatitis C virus entry have focused on the number of viral envelope proteins E2 that must bind with the cell surface receptors CD81 for successful entry [61,65]. (In one study, the presence of another cell surface molecule SR-BI has been shown to aid hepatitis C virus entry, but entry without CD81 binding has not been demonstrated [65].) Accordingly, the models rarely admitted multiple entry pathways. Our model considered two independent entry pathways, allowing the elucidation of the synergy achieved by blocking them both. Furthermore, by using suitably designed limiting or extreme scenarios, our analysis brought out conceptually the origins of the synergy both at the single cell and the cell population levels. The resulting findings are likely to apply beyond SARS-CoV-2 because the entry of several other viruses, including other coronaviruses such as SARS-CoV, MERS-CoV, and influenza viruses [23], occurs similarly, via two independent pathways.

Furthermore, our model was able to estimate the relative usage of the two pathways across different cell lines by analysis of available *in vitro* data. We found that the relative usage of the pathways was similar between SARS-CoV-2 and SARS-CoV but varied widely across cell lines, with the usage of the TMPRSS2 pathway varying from ~0% to ~100% on average depending on the cell line. The variability could be due to the different expression levels of the proteases across the cell lines [45–47]. The variability implies that synergy both at the single cell and cell population may be expected *in vivo* from a drug combination targeting the two pathways.

Given a desired efficacy of the combination, arising from considerations, for instance, of achieving a desired decline in viral load [58], optimal drug dosages can be estimated that would maximize synergy while ensuring efficacy, as has been demonstrated in other settings [40,43,52,54,71]. Our model provides a framework that can be readily applied to predict these optimal dosages. The optimization would require knowledge of the distributions of the proteases across cells and tissue types targeted by SARS-CoV-2, which is beginning to be acquired [45,46]. We expect our study to prompt further investigations into the usage of the two pathways by SARS-CoV-2 *in vivo*.

Studies on other coronavirus infections of mice have remained inconclusive on the synergy between TMRPSS2 and Cathepsin B/L inhibitors. In some studies on SARS-CoV, synergy was not observed in mice although it was evident *in vitro* [72,73]. Studies with the coronavirus HCoV229E, among the viruses causing common cold, attributed such differences between *in vitro* and *in vivo* settings to mutations associated with the laboratory adaptation of the virus to cell lines [74,75]. The mutations have been argued to allow Cathepsin B/L usage *in vitro*, while the *in vivo* strains predominantly use TMPRSS2. Yet, successful SARS-CoV infection of TMPRSS2 knockout mice was observed, demonstrating the ability of SARS-CoV and suggesting the ability of SARS-CoV-2 to use Cathepsin B/L *in vivo* [72]. Besides, the proposed laboratory adaptation appears unlikely to have happened with SARS-CoV-2 because the virus isolated from infected individuals was used in the experiments with minimal *in vitro* passaging [21]. Yet, synergy has been seen *in vitro* between camostat mesylate and E-64D with SARS-CoV-2 [21], suggesting that such synergy may also occur *in vivo*.

We note that in addition to TMPRSS2 and Cathepsin B/L, with SARS-CoV-2, unlike SARS-CoV, the use of furin for S protein activation has been suggested [20,46,76,77]. In a recent study, the role of furin in S protein activation of SARS-CoV-2 was argued to be similar to that in MERS-CoV, where furin cleaves S at the S1/S2 site, following which TMPRSS2 cleavage of S is required for entry through the fusion pathway [77]. Thus, furin does not appear to replace TMPRSS2, but instead places an additional requirement, akin to ACE2 binding, for entry through the TMPRSS2 pathway. Further, eliminating the furin cleavage site did not affect entry through the Cathepsin B/L, or endocytosis, pathway. Our prediction of the synergy between TMPRSS2 and Cathepsin B/L inhibitors is thus independent of the role of furin. Further, a recent study has argued that SARS-CoV-2 may utilize other TMPRSS2-related serine proteases to enter target cells with varying efficiencies [78]. Camostat mesylate was found to block all these proteases. Whether other inhibitors of TMPRSS2 also block related proteases remains to be ascertained. Combining Cathepsin B/L inhibitors with highly specific TMPRSS2 inhibitors may permit virus entry via TMPRSS2-related proteases, compromising therapy and limiting the synergy with Cathepsin B/L inhibitors.

In summary, our study identified unexpected synergy between TMPRSS2 and Cathepsin B/L inhibitors against SARS-CoV-2 infection, highlighted its existence in available *in vitro* studies, and elucidated its novel origins at the single cell and cell population level using mathematical modelling. Our findings may have implications for the treatment of COVID-19 using agents blocking host proteases required for SARS-CoV-2 entry. Although we focussed on TMPRSS2 and Cathepsin B/L, our findings apply more broadly to combinations of agents that block the two independent pathways, fusion and endocytosis, of SARS-CoV-2 entry into target cells.

## Methods

### Model of SARS-CoV-2 entry via TMPRSS2 and Cathepsin B/L mediated pathways

We considered *in vitro* experiments where target cells expressing TMPRSS2 and Cathepsin B/L were exposed to virions or pseudotyped viral particles bearing the spike protein, S. Experiments were performed in the absence or presence of inhibitors of TMPRSS2 and Cathepsin B/L and the fraction of infection events measured post virus exposure. We developed the model below to describe the ensuing viral dynamics.

#### Viral dynamics

We recognized that the expression levels of the proteases would vary across cells and affect viral entry into the cells. Following previous approaches [55,61], we divided the cells into subpopulations based on the expression levels of the proteases. We defined *T*_*tc*_ as the subpopulation of target cells expressing TMPRSS2 in a narrow range Δ*n*_*t*_ around *n*_*t*_ copies per cell and Cathepsin B/L in a narrow range Δ*n*_*c*_ around *n*_*c*_ copies per cell. We described the relative susceptibility of these cells to virus entry through the TMPRSS2 pathway, *S*_*t*_, and the Cathepsin B/L pathway, *S*_*c*_, using Hill functions $S_{t}^{}=\frac{{\left(n_{t}^{}\right)}^{h_{t}}}{{\left(n_{t}^{50}\right)}^{h_{t}}+{\left(n_{t}^{}\right)}^{h_{t}}}$ and $S_{c}^{}=\frac{{\left(n_{c}^{}\right)}^{h_{c}}}{{\left(n_{c}^{50}\right)}^{h_{c}}+{\left(n_{c}^{}\right)}^{h_{c}}}$, where *h*_*t*_ and *h*_*c*_ were the Hill coefficients, and $n_{t}^{50}$ and $n_{c}^{50}$ were protease expression levels at which *S*_*t*_ = 0.5 and *S*_*c*_ = 0.5, respectively. Such Hill functional forms have been used to describe the effects of other proteases [79]. The overall susceptibility of the cells, due to entry through both the pathways, was *S*_*tc*_ = *S*_*t*_+*S*_*c*_−*S*_*t*_*S*_*c*_. In the presence of a TMPRSS2 inhibitor, a Cathepsin B/L inhibitor, or both, the susceptibilities were lowered to *S*_*tc*_(*D*_*T*_), *S*_*tc*_(*D*_*C*_), and *S*_*tc*_(*D*_*T*_,*D*_*C*_), respectively, where *D*_*T*_ and *D*_*C*_ were the concentrations of the TMPRSS2 and Cathepsin B/L inhibitors. The following equations described the ensuing viral dynamics:

$$\frac{dT_{tc}}{dt}=(\lambda -\mu )T_{tc}-kS_{tc}^{}T_{tc}V;\;t=1,2,\ldots ,M;\;c=1,2,\ldots ,N$$ (1)

$$\frac{dI_{tc}}{dt}=kS_{tc}^{}T_{tc}V-\delta I_{tc};\;t=1,2,\ldots ,M;\;c=1,2,\ldots ,N$$ (2)

$$\frac{dV}{dt}=p\sum_{c=1}^{N}\sum_{t=1}^{M}I_{tc}-c_{V}V$$ (3)

Here, target cells proliferate and die with rate constants *λ* and *μ*, respectively. Incorporating the carrying capacity of the cell culture did not affect our results (S4 Fig). *k* is the second order rate constant of the infection by free virions, *V*, of target cells expressing excess TMPRSS2, Cathepsin B/L, or both, so that entry is not limited by the proteases, *i*.*e*., for cells with the susceptibility *S*_*tc*_ = 1. *k* is reduced for cells *T*_*tc*_ by the factor *S*_*tc*_, the relative susceptibility. We recognize that the susceptibility could be altered by variations in the expression levels of other factors such as ACE2 or type I interferon-stimulated proteins, as seen in measurements [45,46] and indicated by the substantial variability in the entry efficiency across different cell lines [21,49,80]. Our focus here is on the relative entry efficiency through the two protease-activated pathways. The susceptibilities are thus relative to the maximum entry efficiency when factors other than TMPRSS2 and Cathepsin B/L are limiting. Successful infection leads to the production of the corresponding infected cells *I*_*tc*_. We neglected the proliferation of infected cells following previous models [61,81]. Infected cells die with a rate constant *δ*. Free virions are produced from infected cells at the rate *p* per cell and cleared with the rate constant *c*_*V*_. We focussed on experiments with pseudotyped viruses, with *I*_*tc*_ representing cells into which successful virus entry has occurred. These pseudotyped viruses are replication incompetent and do not produce new virions. We therefore neglected progeny virion production (*p* = 0).

#### Distribution of protease expression levels across cells

We assumed that at the start of infection, the fraction, *ϕ*_*tc*_ = *ψ*_*tc*_(*n*_*t*_,*n*_*c*_)Δ*n*_*t*_Δ*n*_*c*_, of cells belonging to the subpopulation *T*_*tc*_ expressing the proteases in the narrow range Δ*n*_*t*_ and Δ*n*_*c*_ around *n*_*t*_ and *n*_*c*_, respectively, was determined by the joint distribution, *ψ*_*tc*_(*n*_*t*_,*n*_*c*_) = *ψ*_*t*_(*n*_*t*_)*ψ*_*c*_(*n*_*c*_), where $\psi_{t}(n_{t})=\frac{1}{n_{t}\sigma_{t}\sqrt{2\pi }}e^{-\frac{{\left(\ln n_{t}-\bar{n_{t}}\right)}^{2}}{2\sigma_{t}^{2}}}$ and $\psi_{c}(n_{c})=\frac{1}{n_{c}\sigma_{c}\sqrt{2\pi }}e^{-\frac{{\left(\ln n_{c}-\bar{n_{c}}\right)}^{2}}{2\sigma_{c}^{2}}}$ were the independent log-normal distributions of the expression levels of TMPRSS2 and Cathepsin B/L, respectively, across cells with $\bar{n_{t}}$ and $\bar{n_{c}}$ the associated means, and *σ*_*t*_ and *σ*_*c*_ the standard deviations. We divided the target cells into *M*×*N* subpopulations, so that *t* = 1, 2,..,*M* and *c* = 1, 2,..,*N*.

#### Effect of drugs

We assumed that drugs bound their target proteases and prevented their functioning. The susceptibility of cells would thus be reduced and be determined by the protease molecules unbound to the inhibitors. In the presence of a TMPRSS2 inhibitor, we let the abundance of free TMPRSS2, denoted $n_{t}^{f}$, follow $n_{t}^{f}=\frac{\gamma_{T}}{\gamma_{T}+D_{T}}n_{t}^{}$. The susceptibility of cells *T*_*tc*_ to infection through the TMPRSS2 pathway reduced to $S_{t}^{}(D_{T})=\frac{{\left(n_{t}^{f}\right)}^{h_{t}}}{{\left(n_{t}^{50}\right)}^{h_{t}}+{\left(n_{t}^{f}\right)}^{h_{t}}}$, and the overall susceptibility became *S*_*tc*_(*D*_*T*_) = *S*_*t*_(*D*_*T*_)+*S*_*c*_−*S*_*t*_(*D*_*T*_)*S*_*c*_. Similarly, in the presence of a Cathepsin B/L inhibitor, we let the abundance of free Cathepsin B/L, $n_{c}^{f}$, available for mediating virus entry, follow $n_{c}^{f}=\frac{\gamma_{C}}{\gamma_{C}+D_{C}}n_{c}^{}$. The susceptibilities accordingly reduced to $S_{c}^{}(D_{C})=\frac{{\left(n_{c}^{f}\right)}^{h_{c}}}{{\left(n_{c}^{50}\right)}^{h_{c}}+{\left(n_{c}^{f}\right)}^{h_{c}}}$, and *S*_*tc*_(*D*_*C*_) = *S*_*t*_+*S*_*c*_(*D*_*C*_)−*S*_*t*_*S*_*c*_(*D*_*C*_). With both the drugs used simultaneously, the susceptibilities were *S*_*tc*_(*D*_*T*_,*D*_*C*_) = *S*_*t*_(*D*_*T*_)+*S*_*c*_(*D*_*C*_)−*S*_*t*_(*D*_*T*_)*S*_*c*_(*D*_*C*_). Here, *D*_*T*_ was the concentration of the TMPRSS2 inhibitor, *D*_*C*_ that of the Cathepsin B/L inhibitor, and *γ*_*T*_ and *γ*_*C*_ were the inhibition constants representing drug levels that reduced the respective free protease levels by half. These expressions for the free protease numbers followed dose-response relationships that could be derived mechanistically assuming reaction equilibrium of drug-protease binding and species balance constraints [61].

#### Synergy

We solved Eqns. [1–3] to predict the number of infected cells at day 1 post-infection in each subpopulation in the absence of drugs, denoted *I*_*tc*_, in the presence of a TMPRSS2 inhibitor, *I*_*tc*_(*D*_*T*_), in the presence of a Cathepsin B/L inhibitor, *I*_*tc*_(*D*_*C*_), and in the presence of both inhibitors, *I*_*tc*_(*D*_*T*_,*D*_*C*_). From these populations, we estimated the total fractions unaffected by the drugs individually and together as $f_{}^{u}(D_{T})=\frac{\sum_{c=1}^{N}\sum_{t=1}^{M}I_{tc}(D_{T})}{\sum_{c=1}^{N}\sum_{t=1}^{M}I_{tc}}$, $f_{}^{u}(D_{C})=\frac{\sum_{c=1}^{N}\sum_{t=1}^{M}I_{tc}(D_{C})}{\sum_{c=1}^{N}\sum_{t=1}^{M}I_{tc}}$, and $f_{}^{u}(D_{T},D_{C})=\frac{\sum_{c=1}^{N}\sum_{t=1}^{M}I_{tc}(D_{T},D_{C})}{\sum_{c=1}^{N}\sum_{t=1}^{M}I_{tc}}$. The expected fraction unaffected assuming Bliss independence was thus $f_{Bliss}^{u}(D_{T},D_{C})=f_{}^{u}(D_{T})f_{}^{u}(D_{C})$, and the extent of Bliss synergy, $\beta_{Bliss}^{}=f_{Bliss}^{u}(D_{T},D_{C})-f_{}^{u}(D_{T},D_{C})$. Thus,

$$\beta_{Bliss}^{}=\frac{\sum_{c=1}^{N}\sum_{t=1}^{M}I_{tc}(D_{T})}{\sum_{c=1}^{N}\sum_{t=1}^{M}I_{tc}}\times \frac{\sum_{c=1}^{N}\sum_{t=1}^{M}I_{tc}(D_{C})}{\sum_{c=1}^{N}\sum_{t=1}^{M}I_{tc}}-\frac{\sum_{c=1}^{N}\sum_{t=1}^{M}I_{tc}(D_{T},D_{C})}{\sum_{c=1}^{N}\sum_{t=1}^{M}I_{tc}}.$$ (4)

To estimate the extent of synergy arising from the effects of the drugs at the single cell level, $\beta_{Bliss}^{cell}$, we performed the same calculations as above but assuming a homogeneous cell population.

Identifying the synergy at the cell population level was more involved. It required deriving ways to decouple single cell level synergy from the cell population level synergy. We accomplished it for single round infection assays, which we describe in S3 Text. Accordingly, the cell population synergy was given by the following expression:

$$\beta_{Bliss}^{popn}=\frac{1}{{\left[\sum_{c=1}^{N}\sum_{t=1}^{M}\phi_{tc}(S_{t}^{}+S_{c}^{}-S_{t}^{}S_{c}^{})\right]}^{2}}\left[\begin{array}{c} \left[\sum_{c=1}^{N}\sum_{t=1}^{M}\phi_{tc}(S_{t}^{}-S_{t}^{}(D_{T}))(1-S_{c}^{})\right]\left[\sum_{c=1}^{N}\sum_{t=1}^{M}\phi_{tc}(S_{c}^{}-S_{c}^{}(D_{C}))(1-S_{t}^{})\right]\\ -\sum_{c=1}^{N}\sum_{t=1}^{M}\phi_{tc}^{2}(S_{t}^{}-S_{t}^{}(D_{T}))(1-S_{t}^{})(S_{c}^{}-S_{c}^{}(D_{C}))(1-S_{c}^{}) \end{array}\right].$$ (5)

### Estimates of the relative usage of the entry pathways

We recognized from our analysis above that data from single round infection assays or, equivalently, assays using pseudotyped viruses could be used to estimate the relative usage of the two pathways for entry. We achieved this by linking the fraction of infection events unaffected by drugs and the mean susceptibilities of the populations as follows. Recall that the susceptibility of the cell subpopulation *T*_*tc*_ is *S*_*tc*_ = *S*_*t*_+*S*_*c*_−*S*_*t*_*S*_*c*_, the latter equal to the fraction of the subpopulation infected on average in the absence of drugs in a single round assay. Accordingly, the total fraction of cells infected in the absence of drugs would be $f^{\inf}=\sum_{c=1}^{N}\sum_{t=1}^{M}\phi_{tc}S_{tc}^{}=\sum_{c=1}^{N}\sum_{t=1}^{M}\phi_{tc}(S_{t}^{}+S_{c}^{}-S_{t}^{}S_{c}^{})=\bar{S_{t}^{}}+\bar{S_{c}^{}}-\bar{S_{t}^{}}\cdot \bar{S_{c}^{}}$, where we defined the mean susceptibilities of the populations to entry through the TMPRSS2 pathway, $\bar{S_{t}^{}}=\sum_{t=1}^{M}\phi_{t}S_{t}^{}$, and that through the Cathepsin B/L pathway, $\bar{S_{c}^{}}=\sum_{c=1}^{N}\phi_{c}S_{c}^{}$, and used the independence of the distributions of the expression levels of the proteases, *ϕ*_*tc*_ = *ϕ*_*t*_*ϕ*_*c*_.

Following the arguments above, the fractions infected in the presence of drugs were similarly $f^{\inf}(D_{T})=\bar{S_{t}^{}(D_{T})}+\bar{S_{c}^{}}-\bar{S_{t}^{}(D_{T})}\cdot \bar{S_{c}^{}}$, $f^{\inf}(D_{C})=\bar{S_{t}^{}}+\bar{S_{c}^{}(D_{C})}-\bar{S_{t}^{}}\cdot \bar{S_{c}^{}(D_{C})}$, and $f^{\inf}(D_{T},D_{C})=\bar{S_{t}^{}(D_{T})}+\bar{S_{c}^{}(D_{C})}-\bar{S_{t}^{}(D_{T})}\cdot \bar{S_{c}^{}(D_{C})}$, where $\bar{S_{t}^{}(D_{T})}$ and $\bar{S_{c}^{}(D_{C})}$ were the mean susceptilities of the populations in the presence of a TMPRSS2 inhibitor and a Cathepsin B/L inhibitor, respectively. These expressions yielded the fractions unaffected by drugs as:

$$f_{}^{u}(D_{T})=\frac{\bar{S_{t}^{}(D_{T})}+\bar{S_{c}^{}}-\bar{S_{t}^{}(D_{T})}\cdot \bar{S_{c}^{}}}{\bar{S_{t}^{}}+\bar{S_{c}^{}}-\bar{S_{t}^{}}\cdot \bar{S_{c}^{}}}$$ (6)

$$f_{}^{u}(D_{C})=\frac{\bar{S_{t}^{}}+\bar{S_{c}^{}(D_{C})}-\bar{S_{t}^{}}\cdot \bar{S_{c}^{}(D_{C})}}{\bar{S_{t}^{}}+\bar{S_{c}^{}}-\bar{S_{t}^{}}\cdot \bar{S_{c}^{}}}$$ (7)

$$f_{}^{u}(D_{T},D_{C})=\frac{\bar{S_{t}^{}(D_{T})}+\bar{S_{c}^{}(D_{C})}-\bar{S_{t}^{}(D_{T})}\cdot \bar{S_{c}^{}(D_{C})}}{\bar{S_{t}^{}}+\bar{S_{c}^{}}-\bar{S_{t}^{}}\cdot \bar{S_{c}^{}}}$$ (8)

The above (Eqs [6–8]) were three equations in the four unknowns $\bar{S_{t}^{}}$, $\bar{S_{c}^{}}$, $\bar{S_{t}^{}(D_{T})}$, and $\bar{S_{c}^{}(D_{C})}$, and needed an additional constraint for solution. Our goal was to estimate the ratio of $\bar{S_{t}^{}}$ and $\bar{S_{c}^{}}$ using the reported measurements of *f*^*u*^(*D*_*T*_), *f*^*u*^(*D*_*C*_), and *f*^*u*^(*D*_*T*_,*D*_*C*_). For this, experiments with saturating drug levels offered a strategy. We considered experiments where the drug levels used were high enough that they blocked their respective pathways nearly fully. In other words, $\bar{S_{t}^{}(D_{T})}∼\bar{S_{c}^{}(D_{C})}∼0$. A test of this criterion was that a nearly complete block of infection occurred when the drugs were used in combination because the criterion would imply *f*^*u*^(*D*_*T*_,*D*_*C*_)~0 (Eq [8]). Under these circumstances, Eqs [6] and [7] became $f_{}^{u}(D_{T})\approx \frac{\bar{S_{c}^{}}}{\bar{S_{t}^{}}+\bar{S_{c}^{}}-\bar{S_{t}^{}}\cdot \bar{S_{c}^{}}}$ and $f_{}^{u}(D_{C})=\frac{\bar{S_{t}^{}}}{\bar{S_{t}^{}}+\bar{S_{c}^{}}-\bar{S_{t}^{}}\cdot \bar{S_{c}^{}}}$, dividing which yielded $f_{}^{u}(D_{C})/f_{}^{u}(D_{T})=\bar{S_{t}^{}}/\bar{S_{c}^{}}$, so that the relative usage of the TMPRSS2 pathway was given by

$$\frac{\bar{S_{t}^{}}}{\bar{S_{t}^{}}+\bar{S_{c}^{}}}=\frac{f_{}^{u}(D_{C})}{f_{}^{u}(D_{C})+f_{}^{u}(D_{T})}.$$ (9)

### Data

We considered experiments were different cell lines are exposed to SAR-CoV or SARS-CoV-2 pseudotyped virions in the presence or absence of TMPRSS2 and Cathepsin B/L inhibitors, and the fraction of infection events unaffected by the drugs was estimated [21,28]. We extracted the data reported using Engauge Digitizer 12.1 and estimated the extent of synergy using the Bliss index. The data is presented in Table 1 and S2 Table. The cell lines and drugs used are mentioned in the Results section and Tables.

### Model calculations and parameters

The model equations were solved using programs written in MATLAB (MathWorks, Natick, MA) and Mathematica. We chose the target cell proliferation and death rate constants representative of Vero cells, typically used for studying SARS-CoV-2 infections *in vitro*. These have been estimated to be 0.77 d^-1^ and 0.22 d^-1^ [82,83]. The infected cell death rate and the virion clearance rate constants have been chosen to apply to SARS-CoV-2 infections. These have been set to 0.53 d^-1^ and 10 d^-1^ in earlier studies [58]. The virion production rate per infected cell was set to zero mimicking pseudo-typed virus infection. The initial target cell and virion populations, the infection rate constant and Hill coefficients in the susceptibility expressions were drawn from studies on other viral infection kinetics, likely typical of *in vitro* assays [55,84]. The means and standard deviations of protease levels as well as the levels at which the susceptibility of cells to entry is 0.5 remain unknown. These parameters were assumed, mimicking other viruses where available [55], and were varied over a large range to assess the sensitivity of our model predictions to uncertainties in their estimates. Our key conclusions were unaffected by these variations (S1 and S2 Figs). The infection and the extent of inhibition were assessed at 1 d post-infection, mimicking experimental protocols [21]. *M* and *N* have been set to be 40. Parameter values are listed in S1 Table. Any deviations from these values are mentioned in the figure captions.

### Parameter sensitivity

We first tested the sensitivity of model predictions to parameter values by increasing or decreasing model parameter values by 2-fold from the baseline value one at time (S1 Fig). We found that the model predicted synergy over a broad range of parameter values (S1 Fig). We next performed global sensitivity analysis and computed partial rank correlation coefficients (PRCCs) of each model parameter [85]. The predictions of synergy were most sensitive to the mean and variance of the expression levels of the proteases and the expression levels defining half-maximal susceptibility (S2A Fig). Synergy diminished in parameter regimes where one entry pathway dominated the other (S2B Fig).

## Supporting information

S1 Text Analytical expression of synergy at the single cell level. (DOCX)

S2 Text Synergy at the cell population level in the two-subpopulation scenario. (DOCX)

S3 Text Model of single round infection assay. (DOCX)

S1 Table Model parameters and their values. The meanings of the symbols are in the Methods. The effects of variations in these parameter values are in S1 Fig.
(DOCX)

S2 Table Relative usage of TMPRSS2 and Cathepsin B/L pathways of entry. We computed the relative pathway usage from the effects of the individual drugs targeting either TMPRSS2 or Cathepsin B/L pathways on overall infection. Infection was by viruses pseudotyped with either SARS-CoV-2 S or SARS-CoV S protein. The fraction uninhibited when both the drugs are used was nearly zero in all the cases considered, indicating that the criterion required for the application of our model was met (see Methods). The relative usage we estimated is presented in Fig 6.
(DOCX)

S1 FigSensitivity to model parameters.The Bliss synergy predicted by the full model by changing model parameter values from the baseline one at a time. The black bars are predictions using the baseline parameter values in S1 Table. The red and blue bars are predictions with parameter values 2-fold lower and higher than the baseline values, respectively. Other parameters values used in various bars: $\bar{n_{t}}$ = 9.5 (red), 11.5 (black) and 13.5 (blue); $\bar{n_{c}}$ = 9.5 (red), 11.5 (black) and 13.5 (blue). The drug concentrations used are *D*_*T*_/*γ*_*T*_ = *D*_*C*_/*γ*_*C*_ = 10^3^.(TIF)

S2 FigGlobal sensitivity analysis.**(A)** Partial rank correlation coefficients (PRCCs) indicating the sensitivity of the model predictions of Bliss synergy to variations in model parameter values. The predicted Bliss synergy is most sensitive to the mean and variance of the expression levels of the proteases and the expression levels defining half-maximal susceptibility. The drug concentrations used are *D*_*T*_/*γ*_*T*_ = *D*_*C*_/*γ*_*C*_ = 10^3^. PRCCs were computed from 2000 runs. $\bar{n_{t}}$ and $\bar{n_{c}}$ were varied between $\ln\left(\frac{10^{5}}{4}\right)$ and ln(4×10^5^), and the other parameters were varied up to 4-fold above and below their respective baseline values. **(B)** The distribution of Bliss synergy predicted from 2000 runs in A. For this analysis, we adapted the MATLAB codes available on Prof. Denise Kirschner’s website (http://malthus.micro.med.umich.edu/lab/usadata/).(TIF)

S3 FigComparison between numerical predictions and analytical approximations of synergy.**(A, B)** The predicted Bliss synergy at the single cell level for varying TMPRSS2 expression and fixed (*n*_*c*_ = 150,000 copies/cell) Cathepsin B/L expression (A) and for varying Cathepsin B/L expression and fixed (*n*_*t*_ = 100,000 copies/cell) TMPRSS2 expression (C) at two different drug concentrations. Solid lines are predictions of the full model with homogeneous protease expression across cells (same as Fig 4). Dashed lines are predictions of the analytical expression $\beta_{Bliss}^{cell}=(S_{t}-S_{t}^{}(D_{T}))(S_{c}-S_{c}^{}(D_{C}))/{\left(S_{t}+S_{c}-S_{t}S_{c}\right)}^{2}$ derived in S1 Text. Other parameters are the same as in Fig 4.(TIF)

S4 FigCarrying capacity of target cells did not affect predictions of overall Bliss synergy.**(A, B)** The predicted Bliss synergy as function of concentrations of TMPRSS2 inhibitor, *D*_*T*_/*γ*_*T*_, and Cathepsin B/L, *D*_*C*_/*γ*_*C*_, with (black line) and without logistic growth (coloured lines). The equation $\frac{dT_{tc}}{dt}=(\lambda -\mu )T_{tc}\left(1-\frac{\sum_{c=1}^{N}\sum_{t=1}^{M}T_{tc}}{T_{\max}}\right)-kS_{tc}^{}T_{tc}V;\;t=1,2,\ldots ,M;\;c=1,2,\ldots ,N$ was used instead of Eq (1) in the main text to mimic logistic growth. Here, *T*_max_ is the carrying capacity of the cell culture, *T*(0) is the initial number of target cells, *D*_*T*_/*γ*_*T*_ = *D*_*C*_/*γ*_*C*_ = *D*/*γ*, and other parameters are the same as in Fig 3. Note that *T*_max_→∞ reduces the equation above to Eq (1).(TIF)
