# Supplementary material for: Targeting TMPRSS2 and Cathepsin B/L together may be synergistic against SARS-CoV-2 infection
Source: PLoS Comput Biol. 2020 Dec 8;16(12):e1008461. doi: 10.1371/journal.pcbi.1008461 (PMC7748278; doi:10.1371/journal.pcbi.1008461)
Supplement: S1 Text — (DOCX) [file pcbi.1008461.s001.docx]

**S1 Text. Analytical expression of synergy at the single cell level**

To understand our model predictions of synergy at the single cell level (Fig 4) more deeply, we considered a single round of infection, typical of pseudotyped virus assays [1,2]. Because we assumed a homogeneous cell population in our calculations above (Fig 4), we could focus on a single cell. We considered a cell with susceptibilities through the two pathways denoted $S_{t}$ and $S_{c}$, respectively. It followed that the probability of it getting infected in the absence of drugs in a single round of infection would be $S_{tc}=S_{t}+S_{c}-S_{t}S_{c}$. With the drugs, we let the susceptibilities be reduced to $S_{t}(D_{T})\leq S_{t}$ and $S_{c}(D_{C})\leq S_{c}$, respectively. With the TMPRSS2 inhibitor alone, the probability of infection would be $S_{tc}(D_{T})=S_{t}(D_{T})+S_{c}-S_{t}(D_{T})S_{c}$, whereas it would be $S_{tc}(D_{C})=S_{t}+S_{c}(D_{C})-S_{t}S_{c}(D_{C})$ with a Cathepsin B/L inhibitor alone. With the drugs used together, the probability would become $S_{tc}(D_{T},D_{C})=S_{t}(D_{T})+S_{c}(D_{C})-S_{t}(D_{T})S_{c}(D_{C})$. Because the cells were assumed to be identical, the probabilities, when the cell numbers are large, would correspond to the fractions of cells infected in the respective scenarios. Thus, the fraction unaffected by the drugs was given by the ratios, $f^{u}(D_{T})=S_{tc}(D_{T})/S_{tc}$ , $f^{u}(D_{C})=S_{tc}(D_{C})/S_{tc}$ and $f^{u}(D_{T},D_{C})=S_{tc}(D_{T},D_{C})/S_{tc}$. Using the expressions above and simplifying, we obtained the Bliss synergy, $\beta_{Bliss}^{cell}$, to be

$\beta_{Bliss}^{cell}=\frac{(S_{t}-S_{t}(D_{T}))(S_{c}-S_{c}(D_{C}))}{\left(S_{t}+S_{c}-S_{t}S_{c}\right)^{2}}$. (S1)

This expression explained the trends predicted in Fig 4. When the TMPRSS2 expression level was low, for instance, $S_{t}$ was low and so was $S_{t}(D_{T})$, implying a small difference between the two, and hence weak synergy. The same argument applied to Cathepsin B/L expression and inhibition. When both the proteases were expressed in significant amounts, the differences $S_{t}-S_{t}(D_{T})$ and $S_{c}-S_{c}(D_{C})$ became significant and sizeable synergy resulted. With increasing drug levels, the differences were amplified, leading to greater synergy. At very high expression levels of TMPRSS2, $S_{t}$ approached unity and so did $S_{t}(D_{T})$ because the drug could not block a large enough fraction of the protease molecules to lower susceptibility. The difference between $S_{t}$ and $S_{t}(D_{T})$ thus diminished, lowering synergy and explaining the non-monotonic trend in synergy observed with drug levels (Figs 4I and 4J). Indeed, the synergy calculated using Eq. [S1] was in close agreement with the predictions in Fig 4 (see S3 Fig).

**S1 Text References**

1. Hoffmann M, Kleine-Weber H, Schroeder S, Krüger N, Herrler T, Erichsen S, et al. SARS-CoV-2 cell entry depends on ACE2 and TMPRSS2 and is blocked by a clinically proven protease inhibitor. Cell. 2020;181: 1–10. doi:10.1016/j.cell.2020.02.052

2. Kawase M, Shirato K, van der Hoek L, Taguchi F, Matsuyama S. Simultaneous treatment of human bronchial epithelial cells with serine and cysteine protease inhibitors prevents severe acute respiratory syndrome coronavirus entry. J Virol. 2012;86: 6537–6545. doi:10.1128/JVI.00094-12
