# Supplementary material for: Targeting TMPRSS2 and Cathepsin B/L together may be synergistic against SARS-CoV-2 infection
Source: PLoS Comput Biol. 2020 Dec 8;16(12):e1008461. doi: 10.1371/journal.pcbi.1008461 (PMC7748278; doi:10.1371/journal.pcbi.1008461)
Supplement: S2 Text — (DOCX) [file pcbi.1008461.s002.docx]

**S2 Text. Synergy at the cell population level in the two-subpopulation scenario**

To elucidate the origin of synergy at the cell population level, we constructed the extreme scenario comprising two cell subpopulations, one expressing TMPRSS2 alone and the other Cathepsin B/L alone. We let the fraction of cells expressing TMPRSS2 be and the fraction expressing Cathepsin B/L be . Further, we let the expression levels of TMPRSS2 in the first subpopulation and Cathepsin B/L in the second be fixed, so that no heterogeneity existed within the subpopulations. The first subpopulation was thus susceptible to entry only via the TMPRSS2 pathway and thus could respond to TMPRSS2 inhibitors alone. We let the susceptibilities of this subpopulation without and with a TMPRSS2 inhibitor be $S_{t}$ and $S_{t}(D_{T})$, respectively. Similarly, the second subpopulation was susceptible to entry via the Cathepsin B/L pathway alone and responded to Cathepsin B/L but not TMPRSS2 inhibitors. We set the corresponding susceptibilities to $S_{c}$ and $S_{c}(D_{C})$, respectively. Neither subpopulation admitted synergy at the single cell level, for each could be affected by only one of the drugs. The cell population thus exhibited no synergy arising from effects at the single cell level. We now considered subjecting the cell population comprised of the two subpopulations to different treatments.

In the absence of treatment, the total fraction of cells infected in a single round assay would be . With a TMPRSS2 inhibitor alone, the fraction would become , whereas it would be with a Cathepsin B/L inhibitor alone. In the presence of both drugs, the fraction infected would become . The fractions unaffected by the drugs in the different scenarios would be given by the ratios, $f^{u}(D_{T})=f^{\inf}(D_{T})/f^{\inf}$, $f^{u}(D_{C})=f^{\inf}(D_{C})/f^{\inf}$ and $f^{u}(D_{T},D_{C})=f^{\inf}(D_{T},D_{C})/f^{\inf}$. Using these expressions, we derived an expression for the Bliss synergy, now denoted $\beta_{Bliss}^{popn}$ to emphasize its origin in the cell population level. We obtained the synergy, $\beta_{Bliss}^{popn}=f^{u}(D_{T})f^{u}(D_{C})-f^{u}(D_{T},D_{C})$, to be

. (S2)

It followed that this synergy would vanish in the absence of cellular heterogeneity; i.e., when or , implying the presence of just one of the two subpopulations. The synergy was maximum when the two subpopulations were fully susceptible ($S_{t}=S_{c}=1$), fully drug responsive ($S_{t}(D_{T})=S_{c}(D_{C})=0$) and equally populous (). The corresponding maximum synergy was $\beta_{Bliss}^{popn}=1/4$.

This limiting, two subpopulation scenario served to elucidate the origin of synergy at the cell population level. Because of the heterogeneity in the utilization of the entry pathways, neither drug could block the infection of both the subpopulations. Both drugs together, however, could protect the entire cell population.
