# Supplementary material for: Targeting TMPRSS2 and Cathepsin B/L together may be synergistic against SARS-CoV-2 infection
Source: PLoS Comput Biol. 2020 Dec 8;16(12):e1008461. doi: 10.1371/journal.pcbi.1008461 (PMC7748278; doi:10.1371/journal.pcbi.1008461)
Supplement: S3 Text — (DOCX) [file pcbi.1008461.s003.docx]

**S3 Text. Model of single round infection assay**

We considered experiments where virions, such as those pseudo-typed with SARS-CoV-2 S protein, were capable of infecting cells but not producing progeny virions, were used, restricting infection to a single round. We derived expressions to estimate the synergy arising from the single cell level and the cell population level as well as the total synergy as follows.

***Synergy at the single cell level.*** To describe the synergy arising at the single cell level, we considered the subpopulation $T_{tc}$. We employed the definitions of the susceptibilities $S_{tc}$ as in our main model (Methods). In a single round assay, the fractions of cells infected in the absence and presence of drugs, on average, would equal the susceptibility of the cells. Thus, we could write the populations of cells infected in the absence of drugs, $I_{tc}=T_{tc}S_{tc}=T_{tc}(S_{t}+S_{c}-S_{t}S_{c})$, in the presence of a TMPRSS2 inhibitor, $I_{tc}(D_{T})=T_{tc}S_{tc}(D_{T})$, in the presence of a Cathepsin B/L inhibitor, $I_{tc}(D_{C})=T_{tc}S_{tc}(D_{C})$, and in the presence of both, $I_{tc}(D_{T},D_{C})=T_{tc}S_{tc}(D_{T},D_{C})$. Employing these expressions in the definitions of the fractions unaffected by the drugs, $f_{tc}^{u}(D_{T})=\frac{I_{tc}(D_{T})}{I_{tc}}$*,*$f_{tc}^{u}(D_{C})=\frac{I_{tc}(D_{C})}{I_{tc}}$ and $f_{tc}^{u}(D_{T},D_{C})=\frac{I_{tc}(D_{T},D_{C})}{I_{tc}}$, and simplifying, we estimated the Bliss synergy in one subpopulation, $\beta_{Bliss}^{tc}=f_{tc}^{u}(D_{T})f_{tc}^{u}(D_{C})-f_{tc}^{u}(D_{T},D_{C})$, as

$\beta_{Bliss}^{tc}=\frac{(S_{t}-S_{t}(D_{T}))(S_{c}-S_{c}(D_{C}))}{\left(S_{t}+S_{c}-S_{t}S_{c}\right)^{2}}$. (S3)

***Overall synergy.*** We next considered the overall synergy, arising from both the single cell and the cell population level. For this, we estimated the total fractions of cells infected in the absence and presence of drugs, recognizing that in each subpopulation, the fraction would equal the corresponding susceptibility. We thus wrote: , , , and , where the fractions infected within the subpopulations were $f_{tc}^{\inf}=S_{tc}$, $f_{tc}^{\inf}(D_{T})=S_{tc}(D_{T})$, $f_{tc}^{\inf}(D_{C})=S_{tc}(D_{C})$, and $f_{tc}^{\inf}(D_{T},D_{C})=S_{tc}(D_{T},D_{C})$. The total fractions unaffected by the drugs were thus $f^{u}(D_{T})=\frac{f^{\inf}(D_{T})}{f^{\inf}}$, $f^{u}(D_{C})=\frac{f^{\inf}(D_{C})}{f^{\inf}}$, and $f^{u}(D_{T},D_{C})=\frac{f^{\inf}(D_{T},D_{C})}{f^{\inf}}$. The extent of Bliss synergy was then $\beta_{Bliss}=f^{u}(D_{T})f^{u}(D_{C})-f^{u}(D_{T},D_{C})$. Substituting the expressions derived above for the fractions infected in terms of the susceptibilities, performing algebraic manipulations, and simplying yielded

. (S4) ***Synergy at the cell population level.*** Deriving expressions for the synergy at the cell population from first principles was not straight forward. We therefore employed the following route. We recognized that the total synergy was a convolution of the synergy at the single cell and the cell population levels. We thus reasoned that the expression for the total synergy above would reduce to the synergy at the cell population level in the scenario where no single cell level synergy existed. The lack of synergy at the single cell level implied $\beta_{Bliss}^{tc}=(S_{t}-S_{t}(D_{T}))(S_{c}-S_{c}(D_{C}))/\left(S_{t}+S_{c}-S_{t}S_{c}\right)^{2}=0$. Accordingly, the second term in the numerator of the expression for the overall synergy, $\beta_{Bliss}$, above dropped. Further, in the first term, the elements corresponding to the same values of *t* and *c* in the two summations also dropped. The resulting expression could thus be written as,

, (S5)

yielding the synergy at the cell population level from single round assays. (Note that the second term in the numerator here is to eliminate parts from the first term that have the same values of *t* and *c* in the two summations.)

We verified that the latter expression reduced to the cell population level synergy that we derived from first principles in the limiting scenario where only two subpopulations existed, one expressing TMPRSS2 and the other Cathepsin B/L (S2 Text). For this, we let the first subpopulation express TMPRSS2 and no Cathepsin B/L and the second vice versa. We thus let $t\in\{1,2\}$ and $c\in\{1,2\}$ and set , , and . Thus, $T_{11}$ and $T_{22}$ were the subpopulations expressing TMPRSS2 alone and Cathepsin B/L alone, respectively. We let their susceptibilities in the absence of drugs be $S_{11}=S_{t}$ and $S_{22}=S_{c}$. In the presence of a TMPRSS2 inhibitor, the susceptibility $S_{11}(D_{T})=S_{t}(D_{T})$, whereas $S_{22}$ would remain unchanged. The opposite happened with a Cathepsin B/L inhibitor. With this description, the expression above for $\beta_{Bliss}^{popn}$ reduced to , identical to the expression we derived from first principles (see Text S2).

We therefore employed the above expression for $\beta_{Bliss}^{popn}$ (Eq. (5) in the main text) to estimate the cell population level synergy in our heterogeneous cell population. The expression, however, was restricted to single round assays. Decoupling the two synergies in multiple round assays was not possible.
