# Supplementary material for: Targeting TMPRSS2 and Cathepsin B/L together may be synergistic against SARS-CoV-2 infection
Source: PLoS Comput Biol. 2020 Dec 8;16(12):e1008461. doi: 10.1371/journal.pcbi.1008461 (PMC7748278; doi:10.1371/journal.pcbi.1008461)
Supplement: S1 Table — The meanings of the symbols are in the Methods. The effects of variations in these parameter values are in S1 Fig. (DOCX) [file pcbi.1008461.s004.docx]

**S1 Table: Model parameters and their values.** The meanings of the symbols are in the Methods. The effects of variations in these parameter values are in S1 Fig and S2 Fig.

| ***Parameter*** | ***Description*** | ***Values*** | ***Ref.*** |
| --- | --- | --- | --- |
| $\lambda$ | Target cell proliferation  rate constant | 0.77 d^-1^ | [82] |
| $\mu$ | Target cell death rate  constant | 0.22 d^-1^ | [83] |
| $\delta$ | Infected cell death  rate constant | 0.53 d^-1^ | [58] |
| *c_V_* | Virion clearance rate constant | 10 d^-1^ | [58] |
| *k* | Infection rate constant of cells with excess proteases | 1x10^-4^ ml ffu^-1^ d^-1^ | [55] |
| $T\left(0\right)$ | Initial target cells | 1x10^5^ cell ml^-1^ | [55] |
| $V\left(0\right)$ | Initial viral titre | 1x10^4^ ffu ml^-1^ | [55] |
| $t_{d}$ | Time of assessment | 24 h | [21] |
| $h_{t}$ | Hill coefficient | 4 | [55] |
| $h_{c}$ | Hill coefficient | 4 | [55] |
| $\overline{n_{t}}$ | Mean of TMPRSS2 distribution | 11.5 | Assumed |
| $\overline{n_{c}}$ | Mean of Cathepsin B/L distribution | 11.5 | Assumed |
| $n_{t}^{50}$ | TMPRSS2 expression at which *S_t_* = 0.5 | 1.2 x 10^5^ copies/cell | Assumed |
| $n_{c}^{50}$ | Cathepsin B/L expression at which *S_c_* = 0.5 | 2.4 x 10^5^ copies/cell | Assumed |
| $\sigma_{t}$ | Standard deviation of TMPRSS2 distribution | 1 | [55] |
| $\sigma_{c}$ | Standard deviation of Cathepsin B/L distribution | 1 | [55] |

^*We focussed on experiments with pseudotyped viruses that do not replicate and produce new progeny virions. The virion production rate per infected cell,^ *^p^*^, was therefore set to zero.^

^**ffu stands for focus-forming units.^
