# Supplementary material for: Targeting TMPRSS2 and Cathepsin B/L together may be synergistic against SARS-CoV-2 infection
Source: PLoS Comput Biol. 2020 Dec 8;16(12):e1008461. doi: 10.1371/journal.pcbi.1008461 (PMC7748278; doi:10.1371/journal.pcbi.1008461)
Supplement: S2 Table — We computed the relative pathway usage from the effects of the individual drugs targeting either TMPRSS2 or Cathepsin B/L pathways on overall infection. Infection was by viruses pseudotyped with either SARS-CoV-2 S or SARS-CoV S protein. The fraction uninhibited when both the drugs are used was nearly zero in all the cases considered, indicating that the criterion required for the application of our model was met (see Methods). The relative usage we estimated is presented in Fig 6. (DOCX) [file pcbi.1008461.s005.docx]

**S2 Table: Relative usage of TMPRSS2 and Cathepsin B/L pathways of entry.** We computed the relative pathway usage from the effects of the individual drugs targeting either TMPRSS2 or Cathepsin B/L pathways on overall infection. Infection was by viruses pseudotyped with either SARS-CoV-2 S or SARS-CoV S protein. The fraction uninhibited when both the drugs are used was nearly zero in all the cases considered, indicating that the criterion required for the application of our model was met (see Methods). The relative usage we estimated is presented in Fig 6.

| ***Infection*** | ***Cell line*** | ***Drugs*** | ***f^u^*(*D_T_*)** | ***f^u^*(*D_C_*)** | ***f^u^*(*D_T_, D_C_*)** | ***Relative usage of TMPRSS2 pathway (%)*** | ***Ref.*** |
| --- | --- | --- | --- | --- | --- | --- | --- |
| SARS-2-S | Vero-TMPRSS2 | Camostat (*D_T_*),  E-64d (*D_C_*) | 0.43 | 0.79 | 0.08 | 64.8 | [21] |
| SARS-2-S | Caco-2 | Camostat (*D_T_*),  E-64d (*D_C_*) | 0.11 | 0.6 | ~0.01 | 84.5 | [21] |
| SARS-2-S | 293T-ACE2 | Camostat (*D_T_*),  E-64d (*D_C_*) | ~1 | ~0.03 | ~0.03 | 3 | [21] |
| SARS-2-S | Calu-3^*^ | Camostat (*D_T_*) | ~0.03 | nd^#^ | nd^#^ | 97 | [21] |
| SARS-S | Vero-TMPRSS2 | Camostat (*D_T_*),  E-64d (*D_C_*) | 0.53 | 0.8 | ~0.02 | 60.2 | [21] |
| SARS-S | Caco-2 | Camostat (*D_T_*),  E-64d (*D_C_*) | 0.1 | 0.52 | ~0.03 | 83.9 | [21] |
| SARS-S | 293T-ACE2 | Camostat (*D_T_*),  E-64d (*D_C_*) | ~1 | ~0.03 | ~0.02 | 3 | [21] |
| SARS-S^*^ | Calu-3^*^ | Camostat (*D_T_*) | ~0.02 | nd^#^ | nd^#^ | 98 | [21] |
| SARS-S | HeLa-ACE2-TMPRSS2 | Camostat (*D_T_*),  EST (*D_C_*) | 0.42 | 0.66 | 0.06 | 61.1 | [28] |
| SARS-S | HeLa-ACE2-TMPRSS2 | Camostat (*D_T_*),  Bafilomycin (*D_C_*) | 0.42 | 0.6 | 0.08 | 58.8 | [28] |

^*^For Calu-3 cells, camostat mesylate achieved ~100% inhibition alone, implying ~100% usage of the TMPRSS2 pathway.

^#^nd – not determined.
