# Supplementary figures and images for: Targeting TMPRSS2 and Cathepsin B/L together may be synergistic against SARS-CoV-2 infection
Source: PLoS Comput Biol. 2020 Dec 8;16(12):e1008461. doi: 10.1371/journal.pcbi.1008461 (PMC7748278; doi:10.1371/journal.pcbi.1008461)

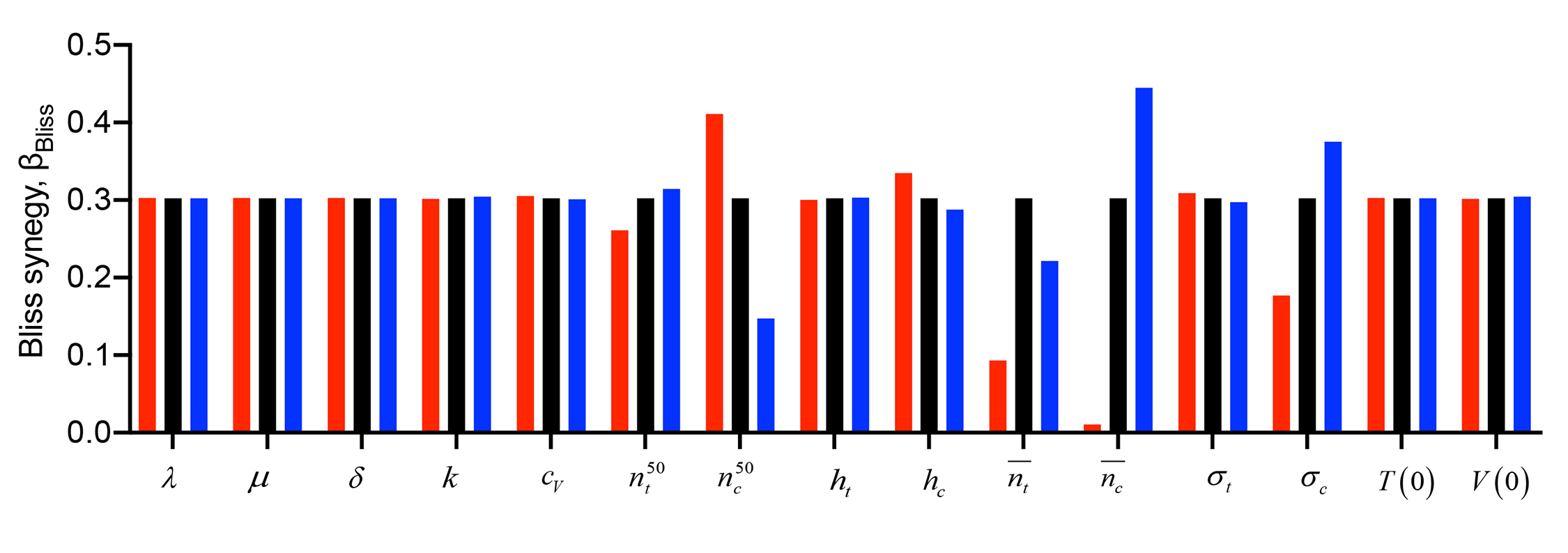

Supplement: S1 Fig — The Bliss synergy predicted by the full model by changing model parameter values from the baseline one at a time. The black bars are predictions using the baseline parameter values in S1 Table. The red and blue bars are predictions with parameter values 2-fold lower and higher than the baseline values, respectively. Other parameters values used in various bars: nt¯ = 9.5 (red), 11.5 (black) and 13.5 (blue); nc¯ = 9.5 (red), 11.5 (black) and 13.5 (blue). The drug concentrations used are DT/γT = DC/γC = 103. (TIF) [file pcbi.1008461.s006.tif]

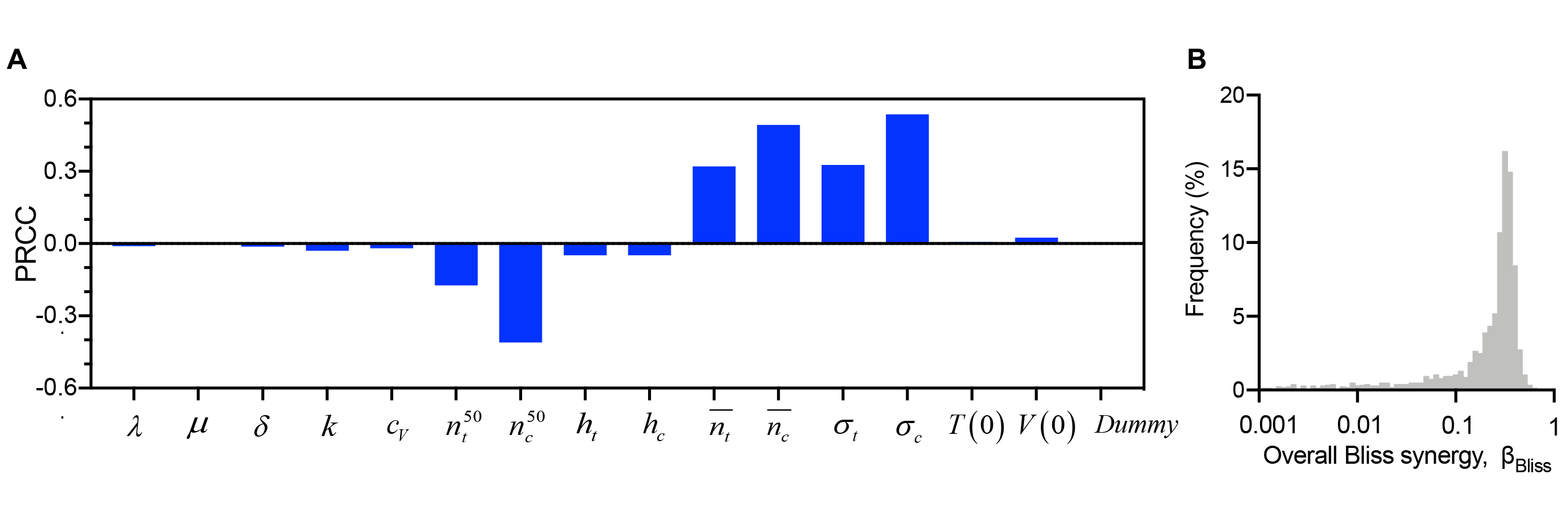

Supplement: S2 Fig — (A) Partial rank correlation coefficients (PRCCs) indicating the sensitivity of the model predictions of Bliss synergy to variations in model parameter values. The predicted Bliss synergy is most sensitive to the mean and variance of the expression levels of the proteases and the expression levels defining half-maximal susceptibility. The drug concentrations used are DT/γT = DC/γC = 103. PRCCs were computed from 2000 runs. nt¯ and nc¯ were varied between ln(1054) and ln(4×10^5^), and the other parameters were varied up to 4-fold above and below their respective baseline values. (B) The distribution of Bliss synergy predicted from 2000 runs in A. For this analysis, we adapted the MATLAB codes available on Prof. Denise Kirschner’s website (http://malthus.micro.med.umich.edu/lab/usadata/). (TIF) [file pcbi.1008461.s007.tif]

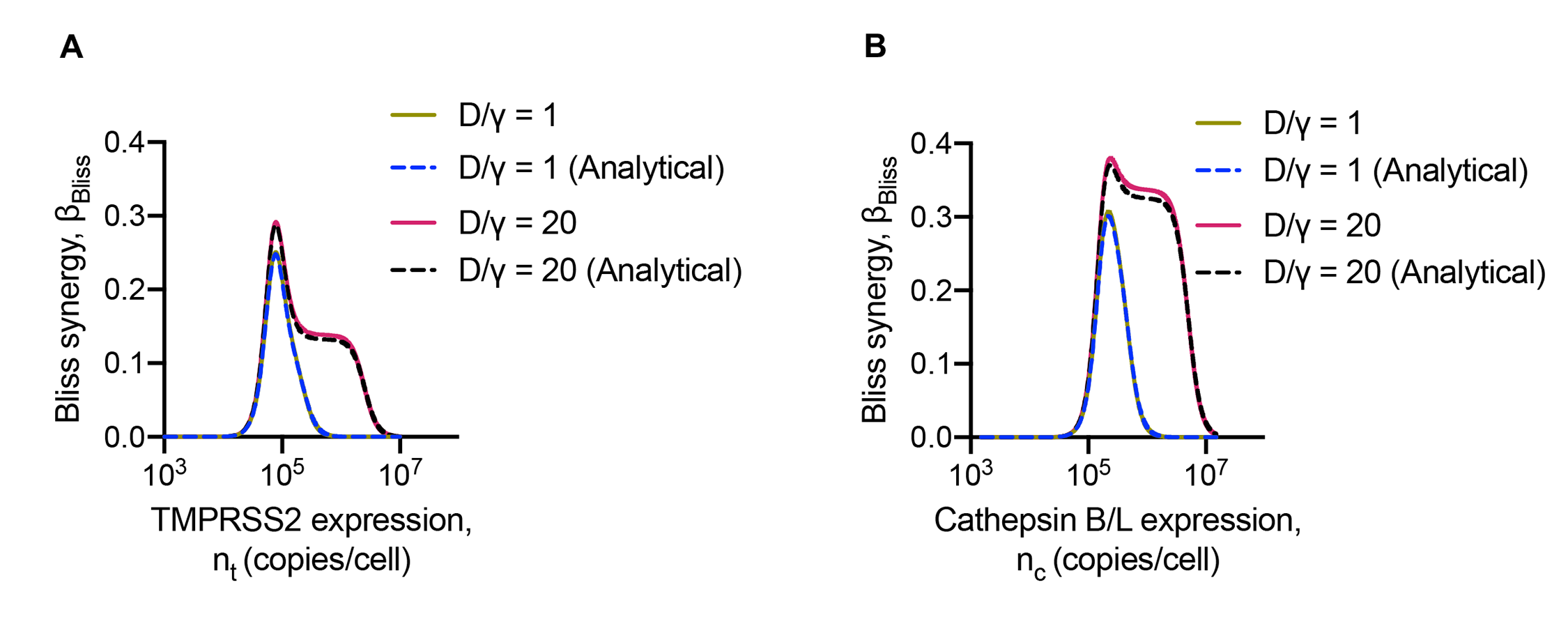

Supplement: S3 Fig — (A, B) The predicted Bliss synergy at the single cell level for varying TMPRSS2 expression and fixed (nc = 150,000 copies/cell) Cathepsin B/L expression (A) and for varying Cathepsin B/L expression and fixed (nt = 100,000 copies/cell) TMPRSS2 expression (C) at two different drug concentrations. Solid lines are predictions of the full model with homogeneous protease expression across cells (same as Fig 4). Dashed lines are predictions of the analytical expression βBlisscell=(St−St(DT))(Sc−Sc(DC))/(St+Sc−StSc)2 derived in S1 Text. Other parameters are the same as in Fig 4. (TIF) [file pcbi.1008461.s008.tif]

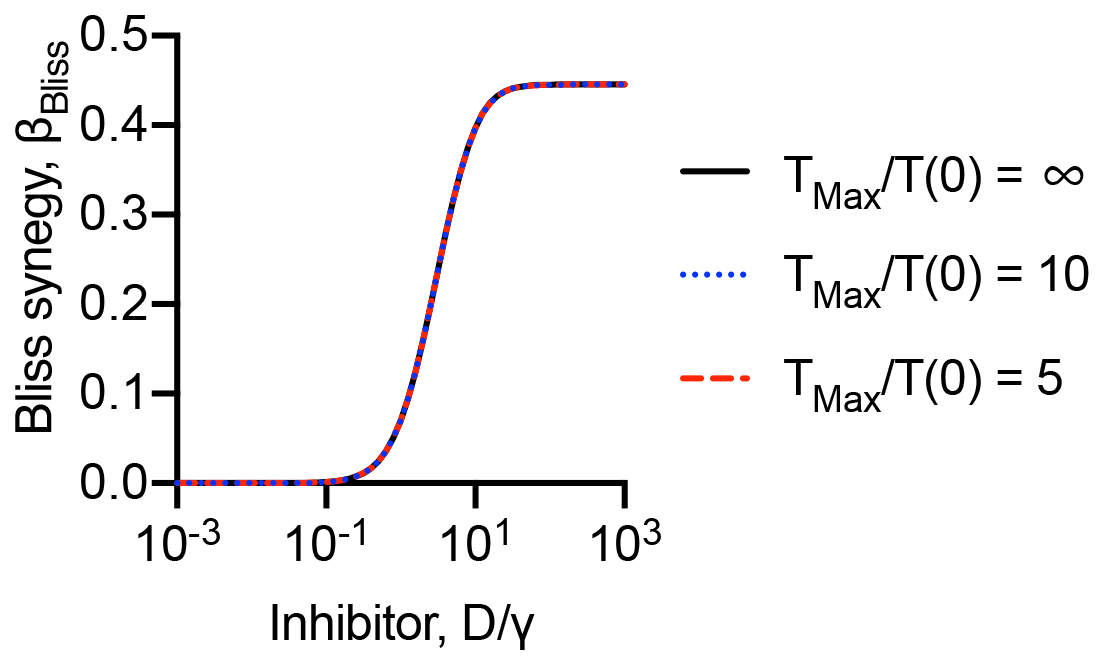

Supplement: S4 Fig — (A, B) The predicted Bliss synergy as function of concentrations of TMPRSS2 inhibitor, DT/γT, and Cathepsin B/L, DC/γC, with (black line) and without logistic growth (coloured lines). The equation dTtcdt=(λ−μ)Ttc(1−∑c=1N∑t=1MTtcTmax)−kStcTtcV;t=1,2,…,M;c=1,2,…,N was used instead of Eq (1) in the main text to mimic logistic growth. Here, Tmax is the carrying capacity of the cell culture, T(0) is the initial number of target cells, DT/γT = DC/γC = D/γ, and other parameters are the same as in Fig 3. Note that Tmax→∞ reduces the equation above to Eq (1). (TIF) [file pcbi.1008461.s009.tif]
